# Natural and artificial sources of genetic variation used in crop breeding: A baseline comparator for genome editing

**DOI:** 10.3389/fgeed.2022.937853

**Published:** 2022-08-22

**Authors:** Jorge Martínez-Fortún, Dylan W. Phillips, Huw D. Jones

**Affiliations:** IBERS, Aberystwyth University, Aberystwyth, United Kingdom

**Keywords:** plant breeding and biotechnology, genome edited crops, genetic variation, traditional breeding, regulation, mutation, genetics, precision-bred organisms

## Abstract

Traditional breeding has successfully selected beneficial traits for food, feed, and fibre crops over the last several thousand years. The last century has seen significant technological advancements particularly in marker assisted selection and the generation of induced genetic variation, including over the last few decades, through mutation breeding, genetic modification, and genome editing. While regulatory frameworks for traditional varietal development and for genetic modification with transgenes are broadly established, those for genome editing are lacking or are still evolving in many regions. In particular, the lack of “foreign” recombinant DNA in genome edited plants and that the resulting SNPs or INDELs are indistinguishable from those seen in traditional breeding has challenged development of new legislation. Where products of genome editing and other novel breeding technologies possess no transgenes and could have been generated *via* traditional methods, we argue that it is logical and proportionate to apply equivalent legislative oversight that already exists for traditional breeding and novel foods. This review analyses the types and the scale of spontaneous and induced genetic variation that can be selected during traditional plant breeding activities. It provides a base line from which to judge whether genetic changes brought about by techniques of genome editing or other reverse genetic methods are indeed comparable to those routinely found using traditional methods of plant breeding.

## 1 Introduction

**FIGURE 1:**
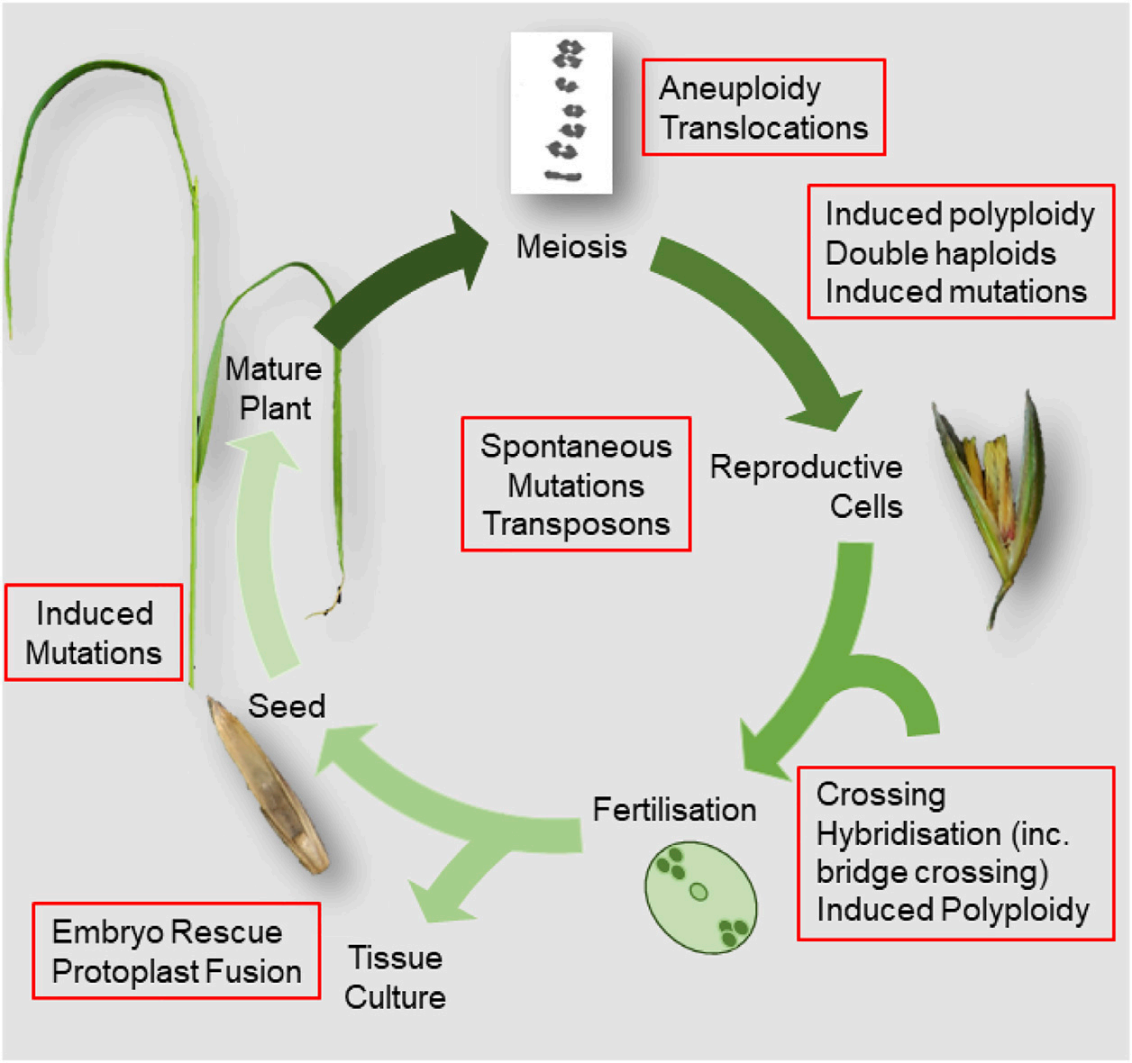
Diagram summarising the flowering plant life cycle showing breeding activities and sources of variation.

The broad aim of plant breeding is to generate new combinations of genetic variants to achieve the desired improvements to a crop’s traits. It is a continuous, iterative process that builds on thousands of years of crop domestication to recombine allelic variation found in existing germplasm, or induced *de novo* by breeding practices, to generate novel genotypes. In this review, traditional plant breeding methods are defined as those that do not use recombinant DNA biotechnologies and that are not regulated under Genetically Modified Organisms (GMO) legislation. These traditional techniques have broadly followed “forward genetic” principles where the desired phenotype (or linked genetic marker) drives selection but where the precise genetic mechanisms that underlie that phenotype are rarely foreknown. For some easy to cross, annual species with abundant natural variation in desired characteristics, the necessary genetic material for specific traits may be readily available within the breeders’ immediate gene pool. However, for other important traits or breeding in vegetatively propagated or self-incompatible crops, the necessary variation can be harder or impossible to access (Figure 1).

Over the last fifty or so years, discoveries and technological developments in nucleotide sequencing, molecular biology, and regenerative tissue culture have introduced the practical use of reverse genetics to complement current practices in plant breeding. New methods for generating variation based on the transfer of defined functional genetic material from one species to another *via* genetic modification has produced many new commercial varieties mainly in maize, cotton, soya and canola. Food and feed derived from genetically modified crops have been commercially available in many countries for decades. However, in others, such as the EU, authorisation for commercial cultivation is effectively blocked. Techniques of genome editing are also being rapidly developed and adopted by many breeding firms. Indeed, food derived from genome edited plants is already publicly available in USA (Menz et al., 2020) and in Japan (Waltz, 2022).

While precise regulatory frameworks and the data requirements for risk assessment for genetic modification vary from country to country, they are broadly established. However, for many crop species it is now relatively facile to generate targeted Single Nucleotide Polymorphisms (SNPs) or Insertion-Deletions (INDELs) using genome editing with no transgenes in the product (Zhang et al., 2018). It is also possible to alter the heritable epigenetic status of specific genetic elements (Mercé et al., 2020). The lack of “foreign” recombinant DNA in these plants confounds the legislative definitions of GMOs in many countries and makes the application of existing biotechnology regulation unworkable for genome edited organisms (Jones, 2015a; b). Some countries have updated or developed new regulatory pathways for gene edited organisms, while others, continue to define all products of genome editing, whether they possess foreign DNA or not, as GMOs (Entine et al., 2021: Schmidt C. et al., 2020).

**FIGURE 2:**
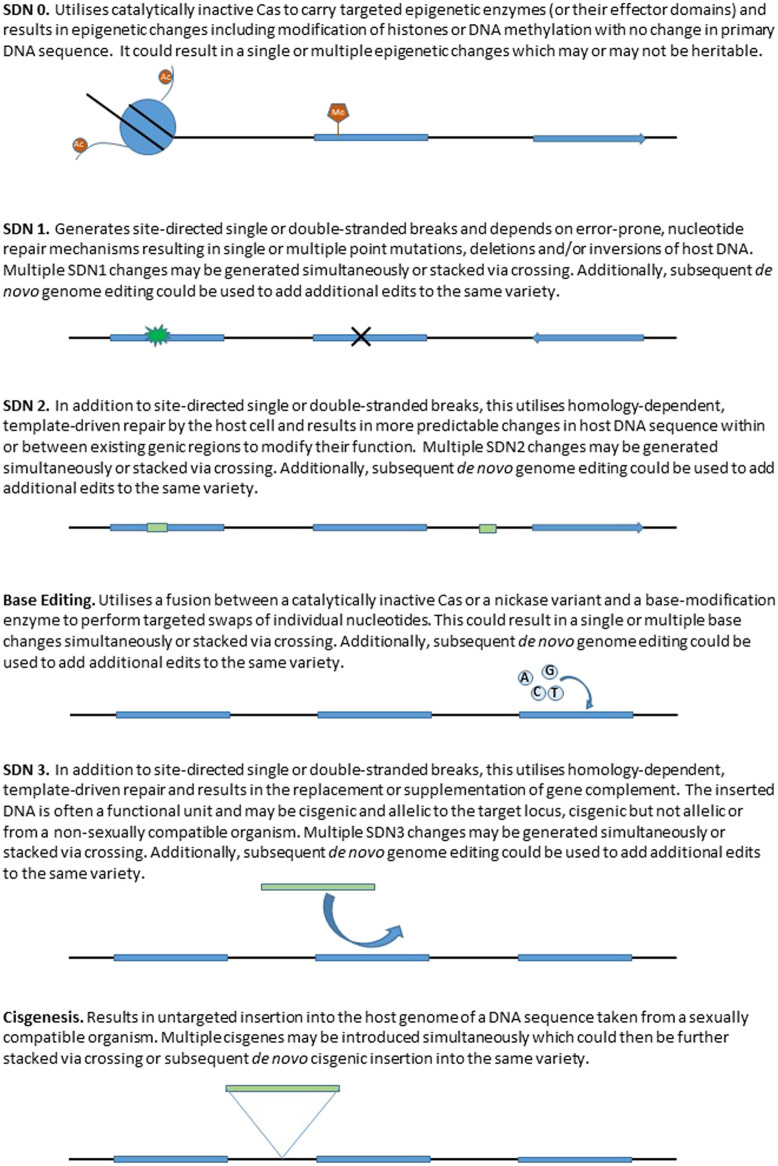
Representation of different site directed nuclease (SDN) genome editing approaches and the resulting changes to the host genome.

A strategy adopted by some authorities is to exclude organisms possessing gene edits that could have been found naturally or produced using traditional, non-biotechnological, breeding methods from GMO regulations. For example, the Japanese Ministry of the Environment has ruled that genome-edited organisms produced by simple double-stranded breaks and repair, so called Site Directed Nuclease-1 (SDN-1) type edits (see Figure 2) are not subject to regulation under the Act on the Conservation and Sustainable Use of Biological Diversity through Regulations on the Use of Living Modified Organisms for implementing the Cartagena Protocol (Cartagena Act), as they are considered similar to those produced by conventional breeding technologies (Tsuda et al., 2019). The Australian Office of the Gene Technology Regulator ruled that genetic edits made without artificially introduced repair templates are no different from changes that occur in nature, and therefore do not pose an additional risk to the environment or human health. However, genome editing technologies that do incorporate template sequences, or that insert other genetic material into the cell (so called SDN-2 or -3 type edits) will continue to be regulated as GMOs (Mallapaty, 2019). In 2018, the Norwegian Biotechnology Advisory Board published a report inviting a public debate on its proposals to accommodate genome editing and discusses the distinction between what genetic changes that can or cannot naturally occur (Bioteknologiradet, 2018). The UK has recently concluded a public consultation exercise which focused on the regulation of gene edited organisms that possessed genetic changes which could have been introduced by traditional breeding (Defra, 2021). The outcomes of this consultation make it clear that a comparison with the types of genetic changes that can be found in existing wild, breeding germplasm, or generated *de novo* by the actions of traditional breeding, and for which regulation already exists, can be a useful first step to define an appropriate regulatory pathway for precision bred organisms.

To date, the different outcomes for genome editing have commonly been designated by regulatory authorities into three categories known as (SDN-1, SDN-2 and SDN-3 (reviewed by Podevin et al., 2013), however these categories are gradually expanding to include more recently developed approaches based on different mechanisms, e.g. modifications to the epigenome (Mercé et al., 2020) and base editing (Azameti and Dauda, 2021). SDN-1 type edits are generated by the formation of targeted Double Strand Breaks (DSBs), which are subsequently repaired, generating SNPs and INDELs at the break site. Such small mutations can generate gene knockouts or induce targeted variation within a gene or associated regulatory sequences, and are equivalent to small sequence changes that arise spontaneously or are induced *via* mutation breeding approaches. SDN-2 type edits exploit the homology directed repair mechanism to introduce small sequence changes using an introduced repair template. This approach is capable of changes equivalent to standard breeding approaches where favourable sequences are combined to generate cultivars that possess enhanced traits. SDN-3 type edits introduce entire genes or longer sequences of novel DNA to a targeted site. In addition, we use the term SDN-0 where deactivated (so-called dead) nucleases are used to perform site directed modification to the epigenome.

This review analyses the types and the scale of spontaneous and induced genetic variation that can be found within and between individuals of a species and which can be selected during traditional plant breeding activities. Thus, it provides a base line from which to judge whether genetic changes brought about by techniques of genome editing or other reverse genetic methods are indeed comparable to those routinely found using traditional methods of plant breeding. It is divided into three main sections with further subdivisions. The section on nucleotide level mutations describes localised and untargeted mutations in DNA sequence resulting in SNPs and INDELs that arise spontaneously from natural biological processes or induced by human intervention. The section on chromosomal level changes describes translocations and rearrangements of chromosomes resulting in the loss, duplication of genes or a change in their position/order. These include changes in individual chromosome structure/number and ploidy levels that can be found in natural populations or artificially induced using a range of traditional breeding techniques. A third section describes breeding methods that modify gene pool barriers to enable wider crosses and utilise otherwise inaccessible genetic variation, both naturally occurring and induced.

The concluding section summarises the main ways that genome editing is currently being used in plant breeding and attempts to compare the types and scales of genetic changes seen in conventional breeding methods to that possible using current genome editing technologies.

### 2.1 Nucleotide level mutations arising from natural processes

Mutations occur spontaneously and accumulate in all cells but those in somatic cells are not passed on to the next generation. In contrast, mutations induced in germline cells that generate the gametes are directly heritable and drive both evolution and the variation available to breeders. Mutations can arise from many natural processes including errors in DNA replication, during DNA repair, by aberrant chromosome behaviour during cell division, or *via* the action of transposable elements.

During replication it is estimated that the error rate of the DNA polymerase is about 1 in 100,000. The inherent proofreading activity of the polymerase complex, and the action of mismatch repair systems, correct the vast majority of these errors, however the few that remain (10^−9^–10^−10^) become fixed mutations in subsequent cell divisions (Mertz et al., 2017). Another source of mutation occurs during prophase I of meiosis when homologous chromosomes undergo crossover formation *via* the reciprocal exchange of non-sister chromatids. The genetic control of the number and position of crossovers is not well understood, but typically most organisms generate between one and three crossover events per chromosome (Fernandes et al., 2018), and these tend to appear at particular genomic hot spots (Drouaud et al., 2006). The crossover process is reliant of the conserved Spo11 enzyme that generates many double stranded DNA breaks (DSBs, Keeney et al., 1997). Although the activity of Spo11 is under tight control, not all DSBs result in reciprocal crossovers and the repair of these non-crossover events can result in intragenic conversion events. Usually, the DSB will be repaired by mechanisms similar to Homologous Recombination (HR) pathways, including resection of the DSB and invasion of a homologous strand (Lawrence et al., 2017). However, in addition to complete and successful crossovers, some DSBs generate errors including non-reciprocal (non-crossover) gene conversion events in one or other homologue. In Arabidopsis between 100 and 200 DSBs are formed per meiotic nucleus, but only around 10 of them are resolve to form crossovers (Copenhaver et al., 1998; Giraut et al., 2011; Salomé et al., 2012; Wijnker et al., 2013), with the remainder resolved as non-crossover products. This process can result in the formation of mutations and contribute to the background level of natural mutations. Various studies have described the mutagenicity of this process in different organisms (Magni and Von Borstel, 1962; Perry and Ashworth, 1999; Lercher and Hurst, 2002; Pratto et al., 2014; Arbeithuber et al., 2015; Rattray et al., 2015). It is known that crossover sites, along with their proximal sequences, possess higher rates of mutation than other genomic regions (Arbeithuber et al., 2015; Yang et al., 2015).

Transposable elements (TE) are repetitive DNA sequences capable of generating dynamic instability and form a large part of many plant genomes. It is estimated that between 10 and 20% of the Arabidopsis genome is composed of TEs (Buisine et al., 2008; Quesneville, 2020) whereas more than 80% of the larger maize genome has similarity to TEs (Baucom et al., 2009; Schnable et al., 2009). There are several classes of TEs that use specific mechanisms to jump between genomic locations. The insertion of TEs into genic coding sequences is particularly mutagenic, often resulting in loss of function alleles.

Although comprehensive data on mutation rates in plants are lacking, mutations appear naturally in the genome at different rates in different species (Hodgkinson and Eyre-Walker, 2011). Arabidopsis and rice are reported to have spontaneous mutation rates of 7 × 10^–9^ and 3.2 × 10^−9^ respectively, base substitutions per site per generation (Ossowski et al., 2010; Yang et al., 2015). The spontaneous mutation rate in maize was reported to be 2.2–3.9 × 10^–8^ per site per generation (Yang et al., 2017).

The majority of these mutations have been observed to be SNPs or short INDELs with 99% of them being shorter than 23 bp, including base pair substitutions and insertion-deletion events (Rice genomes project, 2014). These mutations arise spontaneously due to a range of causes including chemical instability of the nucleobases, copying errors during DNA replication and miss-repair of DSB from naturally occurring mutagens.

### 2.2 Sequence level mutations arising from mutation breeding

For some breeding goals, the available observed variation is insufficient to supply the necessary range of alleles. The observed mutation rates described above can be significantly increased using mutagens, which affect the DNA in different manners (Reviewed in Griffiths et al., 2000; Roychowdhury and Tah, 2013; Oladosu et al., 2016). The induction of genetic variability and appearance of new traits has been, and will continue to be, key for crop development (Novak and Brunner, 1992). One of the mechanisms of mutation induction is the addition of base analogs. These mutagens result in the inclusion of analogs in the DNA sequence followed by mispairing during replication. Examples of these are 5-bromouracil, an analog of thymine which when ionized pairs with guanine (Jacobs, 1969), or 2-amino-purine, an analog of adenine which pairs with cytosine when protonated (Pitsikas et al., 2004).

A similar effect can be obtained using agents that modify an existent base and lead to a specific mispairing, such as alkylating agents. Ethyl-methanesulfonate (EMS) is one of the most common examples, and adds an ethyl group to the nitrogenated bases (Kim et al., 2006). Although it is able to modify all four nucleotides, it has been observed a preference for guanine alkylation, making it pair with thymine instead of cytosine. This agent has a reported mutation rate of one mutation per 3.2 Mb in tomato (Minoia et al., 2010). Other examples of these agents are nitrosoguanidine (MNNG), with effects similar to EMS in both guanine and adenine residues (Goering and Pattee, 1971; Silverman et al., 2001; Wyatt and Pittman, 2006), or methyl-methanesulfonate (MMS), which induces the addition of a methyl group (Lundin et al., 2012). Another example of agents modifying directly the nitrogenated bases is the case of intercalating agents, such as proflavine, acridine orange and other chemicals referred to as ICR compounds. These usually cause single nucleotide INDELs (Hoffmann et al., 2003).

Another pathway for mutagenesis is the induction of base damage. This mechanism blocks any type of base pairing, in contrast with the previous examples that cause mispairing. The lack of pairing leads to a stoppage in replication. The damage will lead to the inclusion of incorrect bases in the sequence. This effect has been described in plants treated with UV light (Stadler, 1928a; Stadler, 1928b), associated mainly with C-T transitions, and transversions, frameshifts, duplications and deletions can also be generated. UV radiation has been observed to induce mutation rates 56 times higher than the usual somatic mutation rate (Kovalchuk et al., 2000). Ionising radiation will also induce DNA damage. This damage has been associated with Reactive Oxygen Species (ROS), which will induce base damage and even strand breaks due to damage in the N-glycosidic bond between the nitrogenated base and the ribose. X-rays are an example of ionising radiation and its effects as a mutagen have been observed in plants (Stadler, 1930). Another example would be the use of γ-rays, which cause short mutations at a frequency of around between 7.5 × 10^–9^ and 9.8 × 10^–6^ (Li J. et al., 2016). Overall, it has been observed that the agents previously described are able to increase mutation rates 56 times in case of UV, 3 times in case of X-rays and 2 times in case of MMS (Kovalchuk et al., 2000), increasing the genetic variation in plant species and leading to a faster appearance of enhanced traits.

A drawback of induced mutagenesis is that only one or other homeologous genes is likely to possess the mutation in the first generation resulting in the need for multiple rounds of backcrossing to combine different mutations and produce homozygosity. Additionally, mutation breeding has limited power in generating favourable dominant alleles and polygenic traits requiring the simultaneous mutations in multiple alleles are unlikely to occur. This is a particular issue in polyploids containing multiple alleles, or other situations where genetic redundancy exists. Also, the screen needed to identify novel traits induced by random by mutation breeding necessitate a very large populations. For difficult to measure traits, it is often unfeasible to exploit this approach.

The use of induced random mutagenesis has been prominent in crop development, with examples of varieties obtained using these methods being cultivated all over the world (Kharkwal and Shu, 2009). In the case of rice, examples such as the “Zhefu 802” variety was obtained by irradiation using γ-rays of “Simei No. 2,” or “Shwewartun” which was obtained by irradiation of the “IR5” variety, and have been widely sown in Asia (Ahloowalia et al., 2004). In barley, the “Diamant” variety was obtained *via* γ-ray irradiation of the “Valticky” variety (Kharkwal and Shu, 2009). Another prominent barley variety, “Golden Promise” was also obtained from γ-ray irradiation of the “Maythorpe” barley variety (Forster, 2004). In North America examples of well-established crops obtained using random mutagenesis include, the “Salinac” variety of bean that was obtained using X rays on the “Navy” variety (Ahloowalia et al., 2004), EMS used to treat the linseed variety “Glenelg” to obtain “Zero,” radio-induced mutagenesis on the wheat variety “Salamanca” was used to obtain “Centauro” and “Bajio Plus” varieties, and in soybean radio-induced mutagenesis on variety “Suaqui 86” was used to obtain “Hector” and “Esperanza” (Green and Dribnenki, 1996). The joint FAO/IAEA mutant variety database lists over 3,000 plant mutant varieties officially released or commercially available around the world (IAEA 2022).

Mutation breeding using external agents described above are well suited to annual crops that reproduce sexually. However, for perennial, vegetatively propagated crops other approaches to generate novel random genetic variation have been used. For example, prolonged periods of tissue culture are known to induce mutations. So-called somaclonal variations are thought to be newly induced mutations arising from the cellular processes of dedifferentiation and differentiation during *in vitro* culture. The causes of genetic instability during tissue culture are not well characterised but it is thought the cellular stresses experience by specialised cells undergoing genetic reprogramming during dedifferentiation contribute to this (Joyce et al., 2003). Although it cannot be excluded that some are pre-existing mutations already present in the explant tissue. Many somaclones have been released as commercial cultivars or varieties, including “While Baron” non-browning potato and heat and salt tolerant flax cultivar “Andro” (Jain, 2001).

### 3.1 Spontaneous chromosome rearrangements and ploidy changes found naturally

Aberrant crossover formation, kinetochore binding, or spindle apparatus during meiosis can lead to a range of changes in chromosome structure, gene order, aneuploidy, and/or ploidy level. In addition to the “normal” meiotic exchanges between homologous chromosomes that generate variation by recombining parental alleles, illegitimate chromosomal translocations or changes in ploidy can also result from aberrant meiotic events. Illegitimate recombination between non-homologous chromosomes can result in deletions, duplications, inversions, and/or translocations. These events can generate new alleles or disrupt the function of others. They can also result in changes to genome size or structure and thus have a profound effect on the variation and evolution of genes and genomes in plants (Cai and Xu, 2007).

#### 3.1.1 Unequal crossovers

Unequal crossing over occurs between the chromatids of homologous chromosomes if they become misaligned during meiosis I, or more rarely between sister chromatids. Although less is known about the direct implications of unequal crossovers in crop plants, these events result in many genetic disorders in humans (Chen et al., 2005). In Arabidopsis, the frequency of meiotic unequal crossing-over that lead to the expansion of a gene cluster and the formation of a novel recombinant locus was approximately 3 × 10^–6^ (Jelesko et al., 1999).

#### 3.1.2 Aneuploidy

The term aneuploidy was first introduced by Täckholm (1922), and describes a deviation from the euploid state *via* the addition or removal of chromosomes (Dyer et al., 1970; Khush, 1973; Sharma, 1990). The occurrence of aneuploidy has been linked to alterations in the chromosomic distribution during cell division, which in plants only occurs in meristematic tissue (mitosis) and reproductive tissue (meiosis). During the mitotic cell cycle, the cell will duplicate its DNA and prepare for cell division. During nuclear division, the chromosomes will condense, align in the metaphasic plate, and be divided in two equal sets of chromosomes. The role of spindles, tubulin and microtubules in this process has been reported in detail (Mazia, 1987). Non-disjunction of chromosomes at anaphase will lead to aneuploid daughter cells. The term “vagrancy” has been used for defining these alterations during mitosis, including metaphasic arrest, chromosome scattering during anaphase, asynchrony of the sets of chromosomes reaching the poles of the cell (forwards/laggards), or non-disjunction of the centromere leading to diplochromosomy, disturbed polarity due to split-spindles leading to aberrant numbers of chromosomes in the daughter cells, or binucleation processes. In case of alterations during meiosis, non-congression, meaning lack of alignment of bivalents during metaphase I, or alterations in disjunction of bivalents, have been observed (Sharma, 1990).

Aneuploidy has been observed to occur naturally in several species including cotton, maize, barley, and oat (Sharma, 1990). However, several environmental factors have also been identified to induce aneuploidy, such as agricultural chemicals, drugs, natural and industrial products, together with others such as soil status, pollution in water, pesticides, plant toxins and pathogens, seed age etc. (Sharma, 1990; Fuchs et al., 2018). Aneuploidy has been linked with the appearance of natural polyploidies as a precursor (Xiong et al., 2011; Chester et al., 2012; Zhang et al., 2013; Wu et al., 2018) and have been observed to induce new variability and phenotypes in different plants (Torres et al., 2008; Veitia et al., 2008), both as an aneuploid (Sheltzer and Amon, 2011) or after recovering its euploidy (Henry et al., 2010; Gao et al., 2016).

#### 3.1.3 Polyploidy

Polyploidy is common in plants and has been a key to the evolution and domestication of many crops. In addition, many crops arising from polyploidy have become commercially relevant (Sattler et al., 2016).

Polyploid species often show increased vigour compared to their diploid relatives and artificial polyploidy has been an attractive goal for plant breeders. Polyploidy arises as the result of total non-disjunction of chromosomes during mitosis or meiosis. Changes in chromosome content involving full duplications of the genetic material occur naturally in plants at different rates, from 30 to 35% (Stebbins, 1971) to 70% (Masterson, 1994), and has been related to different factors such as temperature, herbivory, wounding, water deficit and nutrients shortage (Ramsey and Schemske, 1998). It has also been related to speciation events in plants (Renny-Byfield and Wendel, 2014).

Polyploids can be divided into those resulting from duplication of genetic content within the same species (autopolyploidy) and those resulting from interspecific hybridisations that possess at least one complete diploid set of chromosomes derived from each parental species (allopolyploidy). The effects and causes of polyploidy in plants have been described and reviewed (Sattler et al., 2016). However, different species respond differently to polyploidisation (Dewey, 1979). One of the effects observed has been the “Gigas effect” (Sattler et al., 2016), meaning an increase in cell size and larger organs. This has been proven particularly useful for ornamental crop breeding (Schifino Wittmann and Dall ' Agnol, 2003). It has also been related to higher tolerance to biotic and abiotic stresses (Levin, 2002). However, the increase in cell size usually implies a reduction in cell division (Stebbins, 1971), which also leads to a reduction in growth and late flowering (Levin, 2002), in addition to meiotic aberrations, such as multivalent formation, during the neo-polyploidisation process that can also lead to infertility of the polyploids (Stebbins, 1971).

An additional consequence of polyploidy is genome redundancy, which implies that deleterious alleles can be “buffered” by other copies of the genome (Soltis and Soltis, 2000; Comai, 2005). This redundancy also implies the possibility of increasing gene variation without affecting essential genes, often resulting in subfunctionalisation and neofunctionalisation (Adams and Wendel, 2005). Polyploidy has also been related to higher heterozygosity (Moody et al., 1993; Osborn et al., 2003), which in turn have been related to better performance in different species such as maize (Randolph, 1942), potato (Mendoza and Haynes, 1974) and alfalfa (Katepa-Mupondwa et al., 2002). Subgenome dominance has also been observed in many polyploids, a phenomena where one genome evolves to have a higher gene content and gene expression level, influencing the phenotype of the crop (Edger et al., 2019).

Allopolyploidization has been related to hybrid vigour, also known as heterosis, with results in increased biomass, stature, growth rate, fertility levels and stress tolerance when comparing the hybrid and the original plant (Chen, 2010). Another role for the creation of polyploids has been restoring the fertility of hybrid species, *via* chromosome doubling, (Olsen et al., 2006; Hegarty et al., 2008).

### 3.2 Induced chromosomal structural mutants and ploidy changes

#### 3.2.1 Translocation breeding

To increase the frequency and range of chromosomal deletions, inversions, translocations, and duplications found naturally, it is possible to induce them. One approach is to exploit interspecific crosses or alien introgression lines and screen for useful crossover events during meiosis. Grain crops of the *Triticeae* such as wheat, rye, barley, along with forage grasses, have been a particular focus of chromosome manipulation (Prieto, 2020). Among the *Triticaceae* family, several chromosomes of different species have been described to contain beneficial traits such as biotic stress resistance (Zeller and Hsam, 1983) or yield potential (Rajaram et al., 1983). These include species in the genera *Aegilops*, *Agropyron*, *Triticum* and *Secale* (Friebe et al., 1996). However, some of these chromosomes carry other genes that are not advantageous. In order to reduce the number of deleterious genes transferred, Sears (1956; 1993) proposed the translocation of chromosomic elements between *Aegilops* and wheat by irradiation of the pollen prior to crossing. This approach has been used for several translocations in wheat with rye, the latter being the most common source of translocations (Rabinovich, 1998). An example of these was the 1BL/1RS translocation, which has been incorporated into a substantial number of commercial wheat varieties. The short arm of rye chromosome 1 transferred much needed resistance to stem, stripe and leaf rusts, powdery mildew, and increased yield (Zeller, 1973). The same chromosome fragment was also utilised in the 1AL/1RS translocation of wheat, providing stem rust, powdery mildew and greenbug resistance (Zeller and Hsam, 1983). Other rye chromosomes, such as 3R and 7R, have also been used in wheat translocations but were of less commercial importance (Jung and Seo, 2014). Oat (*Avena sativa*) has also benefitted from this approach when receiving a translocation from *Avena barbata* (Aung and Thomas, 1978).

#### 3.2.2 Induced polyploidy and aneuploidy

Several techniques have been used for inducing polyploids in plants. For the induction of chromosome doubling in somatic cells, temperature has been used to induce ploidy changes in maize (Randolph, 1932), rye and wheat (Dorsey, 1936). Polyploidy is also induced using colchicine, an inhibitor of spindle formation (Blakeslee and Avery, 1937; Planchais et al., 2000). However, its affinity for plant tubulins has been found to be low (Dhooghe et al., 2011), pushing for the introduction of other compounds with similar effects. Examples of these are the dinitroaniline and phosphoric amide-based herbicides (Planchais et al., 2000). In some cases, somatic doubling is not possible (Dewitte et al., 2012), requiring sexual polyploidization (Ramanna and Jacobsen, 2003). This is based on the fusion of unreduced reproductive cells, having the effects observed in polyploids and benefitting from increased variability due to the possibility of recombination and high level of heterozygosity (Ramsey and Schemske, 1998; Peloquin et al., 1999). This technique has been facilitated by different treatments such as temperature, nitrous oxide, anti-tubulin agents, EMS, in addition to gene silencing using RNAi or virus induced gene silencing (VIGS) (Dewitte et al., 2012). This approach has been used in different plants of commercial interest (Peloquin et al., 1999; Ramanna and Jacobsen, 2003).

Several species of commercial significance have been obtained through hybridizations using some of these methods. Triticale (*Triticosecale*) is one of the most commercially relevant hybrids (Ayalew et al., 2018). Obtained as an allopolyploid between wheat (*Triticum aestivum*, 2n = 42) and rye (*Secale cereale,* 2n = 14), it has been developed in different polyploid versions, tetraploid (2n = 14, Łapiński, 2002; Mcgoverin et al., 2011) hexaploid (2n = 42, Merker, 1975; Oettler, 2005) and octoploid (2n = 56, Oettler et al., 1991; Furman et al., 1997; Oettler, 2005), with the hexaploid version being the most commonly cultivated. This is an example of amphiploidy, where the complete genomes of both parental species are included in the hybrid. To circumvent the sterility induced by this event, a doubling of the genome after the hybridization was required. When compared to the progenitors, Triticale has positive properties from both parents, having the adaptative prowess of rye with the yield and grain quality from wheat. However, it is currently more commonly harvested as a crop to be ensiled and used as animal feed.

Another example is the tobacco hybrid *Nicotiana digluta*. *Nicotiana tabacum* (2n = 14) has been crossed with *N. glutinosa* (2n = 12). The sterility of the amphiploid hybrid obtained, *N. digluta* (2n = 36), was overcame by chromosome doubling using colchicine. The hybrid has been backcrossed with *N. tabacum* to obtain a mosaic virus resistant line (Valleau, 1949). A similar approach was used for the synthesis of Raphannobrassica, obtained by crossing *Raphanus sativus* (2n = 36) and *Brassica oleracea* (2n = 18), either by crossing tetraploid forms of the parentals or by crossing and doubling the ploidy levels of the offspring using colchicine (McNaughton, 1973). Vegetatively propagated crops that are often sterile due to the miss-segregation of their triploid genome have been recreated to develop new cultivars. For example, improved diploid members of Banana (genus *Musa*) have been crossed to generate seedless triploid varieties (reviewed by Heslop-Harrison and Schwarzacher, 2007).

Aneuploidy has been used for decades to induce variations and to provide breeding material in plants of commercial relevance. For example, in the wheat cultivar Chinese Spring, there are complete sets of nullisomics, monosomics, trisomics and tetrasomics, along with various combinations of telocentrics (Sears, 1954). Aneuploidy in the wheat cultivar Chinese Spring have been widely used to study the chromosome location of rust pathogen resistance genes (Kimber, 1977).

#### 3.2.3 Doubled haploids

Breeding programs often seek to rapidly fix desirable variation found in individuals. Techniques such as the use of double haploid lines have been utilised to obtain homozygous plants faster than using traditional breeding methods (Chen et al., 2011).

In natural conditions, plants can reproduce using an unconventional reproductive mechanism which can result an embryo that only contains the genome from one parent (Schwander and Oldroyd, 2016). In nature, this can occur either by fusion of the reproductive cells followed by the elimination of one of the genomes (Rieger et al., 2012) or by fertilization of a non-nucleate egg (Pigneur et al., 2012). When the genetic material comes from pollen, the term androgenesis is used, whereas when the genetic material comes from the ovary, gynogenesis or parthenogenesis has been used (Kasha and Maluszynski, 2003; Schwander and Oldroyd, 2016). Generally, this implies that the offspring derived from this process will be haploid, although the common individual is usually diploid (Seguí-Simarro, 2010). In those cases, the genome of the haploid offspring will be doubled to form a diploid specimen, forming a completely homozygous plant (Kermicle, 1974). Androgenesis in plants has also been related to hybridization, given meiotic impairment and production of non-nucleate gametes (Waldman, 2008).

These events have been observed to occur spontaneously in nature, with examples reported in Cupressus dupreziana, an African conifer (Pichot and El Maâtaoui, 2000; Pichot and Maataoi., 2000; Abdoun and Beddiaf, 2002; Pichot et al., 2008), Cupressus sempervirens (Pichot et al., 2001; Burt and Trivers, 2006), maize (Kermicle, 1974) and tobacco (Goodsell, 1961; Burk, 1962; Chase, 1969). Examples of hybrids in pepper (Capsicum frutescens, (Campos and Morgan, 1958), chick pea (Cicer arietinum, Mallikarjuna et al., 2005), oilseed rape (*Brassica* napus, Chen and Heneen, 1989), tobacco (Clausen and Lammerts, 1929; Kostoff, 1929; Kehr, 1951; Burk, 1962; Seguí-Simarro, 2010)*,* Texas bluegrass (*Poa arachnifera,* Seguí-Simarro, 2010), *Solanum verrucosum* (Seguí-Simarro, 2010; Seguí-Simarro et al., 2011) and maize (Goodsell, 1961; Chase, 1969; Kermicle, 1974) have been reported.

Different techniques have been used to achieve induced double haploid production. Chase (1952) described a method based on parthenogenesis in maize. The frequency of parthenogenesis has been increased using pollen irradiation before pollination, use of seeds with twin embryos, sparse pollination, wide hybridizations, or alien cytoplasm inductions in hybrids (Kasha and Maluszynski, 2003). Also crosses between wild diploid and cultivated tetraploid potato formed dihaploid potato through parthenogenesis (Chase, 1963). Wide hybridizations can lead to the loss of one of the sets of chromosomes. Kasha and Kao (1970) described a method of haploid production in cultivated barley by crossing with *Hordeum bulbosum*. Choo et al. (2011) reviewed different hybridization-based techniques for double haploid development in barley. Laurie and Bennett (1988) showed similar findings using maize, sorghum, or millet to pollinate wheat. Barclay (1975) reported double haploids obtained from chromosome elimination after hybridization in wheat as well.

Other techniques for the generation of double haploids utilise the treatment and culture of specific tissues, namely reproductive tissues. Ovule culture has been prominent in sugar beet and onion (Zhang et al., 1999). An alternative strategy is the use of temperature-shock treatment in anthers (Seguí-Simarro, 2010), that has been successfully used in *Crepis tectorum* (Gerassimova, 1936) and *Antirrhinum majus* (Ehrensberger, 1948). *In vitro* anther culture has also been utilised to artificially generate double haploids. This process often requires treatment with temperature shock and *in vitro* culture of the anthers, which usually then diploidise autonomously (Guha and Maheshwari, 1964, 1966; Wang et al., 2000). Anther culture (Clapham, 1973; Ouyang et al., 1973; Kao et al., 1991), pollen culture (Datta and Wenzel, 1987) and microspore culture (Datta and Wenzel, 1987; Kao et al., 1991) have been described in barley and wheat (Weyen, 2008), among other species.

More recently, haploid plants were produced using mutants null for a gene expressing the CENH3 centromere protein. Aberrant spindle microtubules result in mis-segregation during meiosis and haploidy which can then be doubled to give fertile dihaploid plants (Ravi and Chan, 2010).

### 4 Overcoming natural species boundaries

#### 4.1 Protoplast fusion

Protoplast fusion has been used to circumvent inter-species crossing incompatibility to obtain heterozygous specimens from vegetatively propagated plants (Schieder, 1982), resulting in plants with beneficial traits (Liu et al., 2005). Several examples of hybrid formation across species, genera, tribe, and families have been obtained (Carlson et al., 1972; Pelletier et al., 1983; Xia, 2009; Ovcharenko et al., 2011). Wang et al. (2013) summarised several crops of commercial relevance developed over the years and grown in China. Other examples include the transfer of cytoplasmic male sterility between *Brassica* species for commercial F1 hybrid production (Cardi and Earle, 1997). Also, interspecific somatic hybrids of *Brassica oleracea* var. botrytis (cauliflower) and *Brassica nigra* (black mustard) for high resistance to black-rot (Wang et al., 2011) and the production of tetraploid chicory (*Cichorium intybus* var. Magdebourg, Rambaud et al., 1992).

The process is based on somatic hybridization (Carlson et al., 1972) that requires protoplast isolation of the two species of interest, adhesion of the interspecific protoplasts and finally cell fusion (Constabel and Cutler, 1985). Cell fusion in plants can occur spontaneously but has been facilitated using different techniques (Withers and Cocking, 1972). Water-soluble polymers, such as polyvinyl-alcohol (Nagata, 1978), dextran, gelatine (Kameya, 1975), lectin (Glimelius et al., 1978) and PEG (Kao and Michayluk, 1974; Wallin et al., 1974), electric stimulation (Sencia et al., 1979; Zimmermann and Scheurich, 1981), liposomes (Nagata et al., 1979), and mineral salts (Michel, 1938; Power et al., 1970; Carlson et al., 1972; Binding, 1974; Melchers and Labib, 1974) have been used to facilitate the production of fused protoplasts.

After the fusion process, the genetic material of the cell can include the complete nuclear genome from both parents, referred to a symmetric fusion, or include partially one of the parental genomes, referred to as asymmetric fusion and often obtained by irradiation treatment before the fusion (Grosser and Gmitter, 2011). Following fusion, *in vitro* culture techniques allow the development of a fertile adult plant.

### 4.2 Bridging crosses

Another approach to extend the breeders gene pool is to use bridge crossing. This technique utilises intermediary species to facilitate the transfer of genes between species where direct crossing is not possible due to differences in ploidy or other blocks to successful pollination or fertilisation. For example, this approach has been used in wheat, where *Aegilops umbeullata* (2n = 14) was crossed first with *Triticum dicoccoides* (2n = 28) and then the hybrid was successfully crossed with *Triticum aestivum* (2n = 42, Rosyara et al., 2019). Incompatible *Nicotiana* species have also been crossed using this approach. *N. sylvestrin* (2n = 24) was used as an intermediate parent to transfer nematode resistance from *N. repanda* (2n = 48) to *N. tabacum* (2n = 48, Burk, 1967). So called “synthetic” hexaploid wheat (AABBD′D′) have been developed by generating a fertile hybrid between tetraploid durum wheat (*Triticum turgidum*, AABB) and diploid wild goat grass (*Aegilops tauschii*, D’D’) and made significant contributions to the CIMMYT wheat breeding programmes over the last 30 years.

Another example was reported in (Buckner et al., 1961), where gene transfer between Italian ryegrass and tall fescue, which are incompatible, was achieved by crossing the Italian ryegrass first with meadow fescue and crossing that hybrid with the tall fescue. Similar studies have reported the usefulness of this technique in different species such as tobacco (Burk, 1967), cowpea (Fatokun, 2000), wheat (Chhuneja et al., 2007) and cotton (Ram, 2014).

### 4.3 Embryo rescue

The bridge crossing technique or wide hybridisations in general can lead to partial infertility or to poor embryo development *in vivo*. This may be due to the lack of endosperm development or long dormancy periods (Reed, 2005). In these cases, *in vitro* culture techniques are useful to support embryo germination and the formation of a fertile adult plant. This process has had different aims, from studies in plant biology to breeding programs (Collins and Grosser, 1984; Bridgen, 1994; Sharma et al., 1996; Haslam and Yeung, 2011; Ramming, 2019). It is used in plants such as cassava (Lentini et al., 2020), where hand crossing can lead to under-developed seeds to increase the chances of producing viable plants in the next generation (Yan et al., 2015). Also, for hybridization between species of cassava and castor bean (Baguma et al., 2019) or some *Aegilops* x *Triticum* crosses, where hybrid embryos are not naturally viable and must be nurtured to maturity *in vitro* (Gill et al., 1981; Miller et al., 1987)].

## 5 Discussion

By generating novel combinations of spontaneous or induced genetic variation, as described above, traditional breeding has successfully selected beneficial traits for food, feed, and fibre crops. The remarkable phenotypic transformation of the wild progenitors into the eventual crop often includes the loss of seed dormancy, loss of toxicity, synchronised seed maturation, altered shattering, higher yield, etc, and is termed “domestication syndrome” (Ross-Ibarra et al., 2007). This extreme population bottleneck defined the genetic variation underpinning these core crop traits and included variation at both the DNA and chromosomal level. The origins of this process date back 10,000 years, although the last century has seen significant technological refinements in both generating and selecting appropriate genetic variation. In the EU alone, this results in the release of around 3,500 new varieties per year (Bruins 2021). A prerequisite for National Listing and the ability to claim breeders’ rights are tests that new crop varieties must undertake for DUS characteristics (distinctiveness, uniformity and stability over generations). Although the EU has no specific pre-market food safety testing for conventionally bred varieties, breeders must comply with relevant aspects of the overarching Regulation EC 178/2002 known as EU “General Food Law,” along with any country-specific laws.

The early success of genome editing in model plants such as tobacco and Arabidopsis has been rapidly replicated in a vast range of crop plants (see reviews by Martínez-Fortún et al., 2017; Gao, 2021). Although some crop species are more amenable and have received more attention for genome editing research than others, its widespread adoption in university and research centres throughout the world has resulted in proof-of-concept examples for most crop species. The EU-Sage database (https://www.eu-sage.eu/) contains over 500 peer-reviewed research articles where genome editing has been used to target market-oriented traits in over 60 different crops with diverse targets including male sterility, altered flowering time, accelerated domestication, better yield, seed/pod shattering, improved plant architecture, herbicide tolerance, resistance to fungi, viruses, bacteria and insects, fatty acid biosynthesis, improved nutritional content, reduction of allergens etc. The most common outcome for genome-edited interventions is gene knockout, *via* either premature stop codons or frame-shift mutations generated by base editing or SDN1 type repair. Although, as knowledge of gene function and interaction improve, SDN-2 and -3 edits, along with base editing and modification of the epigenome, are set to enable designed alteration of enzymes, storage or structural proteins and transcription factors. In addition, the multiplexing of trait enhancement in these crops will become ever more routine as the market for these crops grows, and the technology becomes further refined (Najera et al., 2019; Abdelrahman et al., 2021). Indeed, it is the ability to rapidly multiplex many changes into the same individual without the constraints of linkage drag and multiple back-crossings to a recurrent parent that make genome editing particularly advantageous. However, despite significant interest from researchers and breeders, and the general enthusiasm for the technology from many food chain stakeholders, only a handful of genome-edited foods have been commercialised to date. One reason for this is the country-to-country variation and general uncertainty over how to regulate this technology.

Taking conventional plant breeding as a baseline, it is logical and proportionate for most products of new breeding technologies to not fall within the scope of current GMO regulations. Specifically, those products of genome editing that possess no transgenes, that could have been generated *via* traditional methods and where the nutritional composition of the edible parts falls within the range of commercial reference varieties, should simply follow the conventional route for variety trials and National Listing.

In Table 1, we have used a four-point scale in an attempt to address what outcomes of genome editing could and could not be achieved using traditional means. Broadly, some applications of genome editing can clearly result in a GMO, for instance where functional transgenes from a non-sexually compatible organism are integrated into the host genome in a site-directed manner. Other outcomes of genome editing, such as individual SNPs or minor INDELs, could clearly be found spontaneously in nature or readily be achieved using traditional breeding methods in sexually propagated crops. Although individual edits are clearly achievable by traditional breeding, in many crops, practical limitations would make multiplexing of these individual edits at multiple loci in the same individual a significant challenge. Although, in other crops, the use of automated, high throughput marker assisted selection could make this more facile. Furthermore, the broad spectrum of methods used by plant breeders are not commonly applied to all crops. Often innovations, such as mutation breeding, or high throughput marker assisted selection have yet to be employed in some orphan crops. This is particularly evident in vegetatively propagated crops, such as banana, where methods relying on sexual reproduction are not applicable (Heslop-Harrison and Schwarzacher, 2007). It is inevitable that genome editing techniques will be used to improve such crops, and therein lies the challenge for regulators. It is relatively easy to categorise particular genome edited plants as GMOs, and others as non-GMO organisms that possess individual genetic changes that could be achieved by traditional breeding. However, examples where multiple editing at multiple loci has been used to introduce a novel function, or where editing has been used in a hard-to-breed crop, may be more difficult to categorise. The authors assume that flexibility will be applied so editing in vegetatively propagated species or the proportionate use of multiplexing will not be disadvantaged. Plant breeding has had an impressive history of safety and where genome editing results in changes that could be generated using traditional methods, it is appropriate that the same safety checks apply. However, where genome editing results in marked nutritional changes to food crops that are outside the normal range or generate a totally novel food type that may raise a safety concern, then regulators may require a proportionate, case-by-case risk assessment.

**TABLE 1:** Applications of genome editing that may or may not be possible to achieve using traditional breeding methods. The level of technical difficulty and/or time needed to for different types of genome editing to be replicated using traditional breeding methods are indicated by: **✓✓✓** relatively facile; **✓✓** possible in longer time frames; **✓** technically challenging, laborious or needing much long time frames; × not possible in any reasonable timeframe.

Category of genome editing	Example of genome editing with references		Ease of replication via traditional breeding methods	Justification	Example references
**SDN-0** Non-enzymatically active CRISPR molecules (dCas9) used to direct DNA methylases or acetyltransferases to alter the epigenetic status of targeted genomic locations with no change in the genome sequence.	Altered DNA methylation	Séré and Martin, (2020), Ghoshal et al. (2021)	Although types and locations of epigenetic marks in a plant can vary over space and time, natural epigenetic variation is more frequent than genetic mutations and specific epigenetic status could, in theory, be selected for	Natural epigenetic variation is widespread, heritable and contributes to plant adaptation. Intentionally or not, it will have been selected for (or against) in traditional breeding.	Becker et al. (2011); Schmid et al. (2018); Varotto et al. (2020)
			Epigenetic alteration at single locus ✓✓		
	Altered Histone acetyltransferase activity	Roca Paixão et al. (2019)	Epigenetic alteration at multiple loci in same plant ✓		
**SDN-1/ SDN-2** Site-directed nuclease with or without repair template can readily generate SNPs and INDELs in a specific diploid or polyploid parental genotype. These may be homozygous in the first generation.	Gene knockout / loss of function alleles via premature stop codon, frameshift etc.	Chandrasekaran et al. (2016), Bull et al. (2018)	Loss of function/ SNP / INDEL at a single pre-determined genomic location ✓✓✓	Insertions, deletions, inversions, and duplications of DNA sequences occur throughout the genome. Forward and reverse genetic screening for individuals possessing equivalent mutations in some crops is facile. Traditional methods to combine multiple mutations at different loci using marker assisted selection is also possible in some crop species but more challenging or impossible in vegetatively propagated, perennial, self-incompatible etc crops. The generation of de novo, functional gene sequences via iterative generation/selection of multiple, independent, contiguous mutations, is effectively impossible using current traditional breeding approaches. However, the introgression of one or multiple genes from a crossable species is relatively facile (see below).	Funatsuki et al. (2014); Hasan et al. (2021)
			Loss of function/ SNPs / INDELs at multiple, independent pre-determined genomic locations in same plant ✓✓		
	Gene knockout / loss of function alleles via multiple SDN excision.	Doll et al. (2019), Li et al. (2022)	The generation of novel, functional gene sequences (including cis- or transgenes) via the generation of multiple contiguous mutations ×		
**Base editing** is emerging as a more facile method to generate targeted SNPs in a specific diploid or polyploid parental genotype. Unlike SDN1/2/3, these do not depend on Non-Homologous End Joining or Homologous Recombination and may be homozygous in the first generation.	Targeted nucleotide substitution	Bharat et al. (2020), Molla et al. (2021)	Base edit at a single pre-determined genomic location ✓✓✓	Substitutions of DNA bases occur spontaneously throughout the genome. As above, screening for individuals possessing equivalent mutations is facile in some crops. Traditional methods to combine multiple mutations at different loci using marker assisted selection is also possible in some crop species but more challenging or impossible in vegetatively propagated, perennial, self-incompatible etc crops. The generation of de novo gene sequences by spontaneous substitution and iterative selection of multiple contiguous bases is effectively impossible using current traditional breeding approaches. However, the introgression of one or multiple existing cisgenes is relatively facile.	Wang et al. (2020)
		Li et al. (2017)	Base edits at several, independent pre-determined genomic locations in same plant ✓		
			Multiple base edits in contiguous nucleotide positions to generate a completely novel gene ×		
**SDN-3 / cisgenics** Single or multiple site-directed nucleases with repair template can produce targeted and homozygous whole gene level changes in an elite diploid or polyploid parental genotype to obviate the need for repeated backcrossing.	Allele (cisgene) replacement or *de novo* cisgene addition	Li S. et al. (2016); Schmidt S.M. et al. (2020); Jo et al. (2014)	Single allele (cisgene) replacement or *de novo* cisgene addition ✓✓✓	Sexual crossing results in novel combinations of alleles. Screening for individuals possessing a specific allele is relatively facile using molecular markers. Traditional methods to combine specific alleles at multiple loci using marker assisted selection is also possible in some crop species but more challenging or impossible in vegetatively propagated, perennial, self-incompatible etc crops. Repeated backcrossing to a recurrent parent combined with MAS can result in a single or multiple allele replacement.	Chung et al. (2017); Cho et al. (2019)
			Multiple allele (cisgene) replacements or *de novo* cisgene transfers into the same plant ✓		
	Targeted insertion of multiple cisgenes or transgenes at a single locus using gene editing	Gao et al. (2020)	Simultaneous insertion of multiple genes into a single, segregating locus ×?	The introgression of one or more cisgenic alleles or the repeated, iterative stacking of cisgenes into untargeted locations is possible. However, introgressing multiple cisgenes into a single, *predefined*, genomic landing locus is effectively impossible using current traditional breeding approaches, However, the function of the individual cisgenes is unlikely to be significantly altered by their genomic location.	
	Trait stacking into a predetermined genomic locus	Ainley et al. (2013)	Iterative stacking by adding new genes to an already present and pre-determined ‘safe harbour’ / ‘landing pad’ locus ×?		

Regardless of the exact constraints placed on genome edited crops in the future, it is inappropriate to regulate them as GMOs where the same outcomes can be achieved *via* traditional methods. It is imperative that society benefit from the agricultural and breeding innovations including genome editing. Unlocking the legal constraints around the application of the technology will drive continued improvements in feed and food crops and contribute to securing food supplies in an ever-changing environment.

## References

[B1] Abdelrahman M. Wei Z. Rohila J. S. Zhao K. (2021). Multiplex genome-editing technologies for revolutionizing plant biology and crop improvement. Front. Plant Sci. 12, 721203. 10.3389/fpls.2021.721203 34691102 PMC8526792

[B2] Abdoun F. Beddiaf M. (2002). [Cupressus dupreziana A. Camus: Distribution, decline and regeneration on the tassili n'Ajjer, central sahara]. C. R. Biol. 325, 617–627. 10.1016/s1631-0691(02)01433-6 12187648

[B3] Adams K. Wendel J. (2005). Polyploidy and genome evolution in plants. Curr. Opin. Plant Biol. 8 (2), 135–141. 10.1016/j.pbi.2005.01.001 15752992

[B4] Ahloowalia B. S. Maluszynski M. Nichterlein K. (2004). Global impact of mutation-derived varieties. Euphytica 135 (2), 187–204. Springer. 10.1007/s10725-010-9554-x

[B5] Ainley W. M. Sastry-Dent L. Welter M. E. Murray M. G. Zeitler B. Amora R. (2013). Trait stacking via targeted genome editing. Plant Biotechnol. J. 11 (9), 1126–1134. 10.1111/pbi.12107 23953646

[B6] Arbeithuber B. Betancourt A. J. Ebner T. Tiemann-Boege I. (2015). Crossovers are associated with mutation and biased gene conversion at recombination hotspots. Proc. Natl. Acad. Sci. U. S. A. 112 (7), 2109–2114. 10.1073/pnas.1416622112 25646453 PMC4343121

[B8] Aung T. Thomas H. (1978). The structure and breeding behaviour of a translocation involving the transfer of mildew resistance from *Avena barbata* Pott. into the cultivated oat. Euphytica 27 (3), 731–739. 10.1007/BF00023709

[B9] Ayalew H. Kumssa T. T. Butler T. J. Ma X. F. (2018). Triticale improvement for forage and cover crop uses in the southern great plains of the United States. Front. Plant Sci. 9, 1130. 10.3389/fpls.2018.01130 30127797 PMC6087761

[B10] Azameti M. K. Dauda W. P. (2021). Base editing in plants: Applications, challenges, and future prospects. Front. Plant Sci. 12, 664997. 10.3389/fpls.2021.664997 34386023 PMC8353127

[B11] Baguma J. K. Mukasa S. B. Kawuki R. Tugume A. K. Buttibwa M. Nalela P. (2019). Fruit set and plant regeneration in cassava following interspecific pollination with castor bean. Afr. Crop Sci. J. 27 (1), 99. 10.4314/acsj.v27i1.8

[B13] Barclay I. R. (1975). High frequencies of haploid production in wheat (*Triticum aestivum*) by chromosome elimination. Nature 256 (5516), 410–411. 10.1038/256410a0

[B14] Baucom R. S. Estill J. C. Chaparro C. Upshaw N. Jogi A. Deragon J.-M. (2009). Exceptional diversity, non-random distribution, and rapid evolution of retroelements in the B73 maize genome. PLoS Genet. 5 (11), e1000732. 10.1371/journal.pgen.1000732 19936065 PMC2774510

[B15] Becker C. Hagmann J. Müller J. Koenig D. Stegle O. Borgwardt K. (2011). Spontaneous epigenetic variation in the *Arabidopsis thaliana* methylome. Nature 480 (7376), 245–249. 10.1038/nature10555 22057020

[B16] Bharat S. S. Li S. Li J. Yan L. Xia L. (2020). Base editing in plants: Current status and challenges. Crop J. 8 (3), 384–395. 10.1016/j.cj.2019.10.002

[B17] Binding H. (1974). Fusionsversuche mit isolierten protoplasten von *Petunia hybrida* L. Z. Für Pflanzenphysiol. 72 (5), 422–426. 10.1016/s0044-328x(74)80063-2

[B18] Bioteknologiradet (2018). The gene technology Act – invitation to public debate. The Norwegian Biotechnology Advisory Board, 52.

[B19] Blakeslee A. F. Avery A. G. (1937). Methods of inducing doubling of chromosomes in plants: By treatment with colchicine. J. Hered. 28 (12), 393–411. 10.1093/oxfordjournals.jhered.a104294

[B22] Bridgen M. P. (1994). Some characteristics of perennial and annual ryegrass × tall fescue hybrids and of the amphidiploid progenies of annual ryegrass × tall fescue ^1^ . Crop Sci. 29, 1243–1246. 10.21273/HORTSCI.29.11.1243

[B23] Bruins M. (2021). The 20 most innovative plant varieties of 2020. Eur. Seed J. 8 (2), 19–24.

[B24] Buckner R. C. Hill H. D. Burrus P. B. (1961). Some characteristics of perennial and annual ryegrass × tall fescue hybrids and of the amphidiploid progenies of annual ryegrass × tall fescue 1. Crop Sci. 1, 75–80. 10.2135/cropsci1961.0011183x000100010022x

[B25] Buisine N. Quesneville H. Colot V. (2008). Improved detection and annotation of transposable elements in sequenced genomes using multiple reference sequence sets. Genomics 91 (5), 467–475. 10.1016/j.ygeno.2008.01.005 18343092

[B26] Bull S. E. Seung D. Chanez C. Mehta D. Kuon J. E. Truernit E. (2018). Accelerated *ex situ* breeding of GBSS- and PTST1-edited cassava for modified starch. Sci. Adv. 4 (9), eaat6086. 10.1126/sciadv.aat6086 30191180 PMC6124905

[B27] Burk L. G. (1967). An interspecific bridge-cross: *Nicotiana repanda* through *N. sylvestris* to *N. tabacum* . J. Hered. 58, 215–218. 10.1093/oxfordjournals.jhered.a107591

[B28] Burk L. G. (1962). Haploids in genetically marked progenies of tobacco. J. Hered. 53 (5), 222–226. 10.1093/oxfordjournals.jhered.a107176

[B29] Burt A. Trivers R. (2006). Genes in conflict: The biology of selfish genetic elements. Am. J. Hum. Biol. 18 (5), 727–728. 10.1002/ajhb.20560

[B30] Cai X. Xu S. S. (2007). Meiosis-driven genome variation in plants. Curr. Genomics 8 (3), 151–161. 10.2174/138920207780833847 18645601 PMC2435351

[B31] Campos F. Morgan D. T. (1958). Haploid pepper from a sperm. J. Hered. 49 (4), 135–137. 10.1093/oxfordjournals.jhered.a106786

[B32] Cardi T. Earle E. D. (1997). Production of new CMS *Brassica oleracea* by transfer of `Anand' cytoplasm from *B. rapa* through protoplast fusion. Theor. Appl. Genet. 94 (2), 204–212. 10.1007/s001220050401

[B33] Carlson P. S. Smith H. H. Dearing R. D. (1972). Parasexual interspecific plant hybridization. Proc. Natl. Acad. Sci. U. S. A. 69 (8), 2292–2294. 10.1073/pnas.69.8.2292 16592009 PMC426920

[B34] Chandrasekaran J. Brumin M. Wolf D. Leibman D. Klap C. Pearlsman M. (2016). Development of broad virus resistance in non-transgenic cucumber using CRISPR/Cas9 technology. Mol. Plant Pathol. 17 (7), 1140–1153. 10.1111/mpp.12375 26808139 PMC6638350

[B35] Chase S. S. (1969). Monoploids and monoploid-derivatives of maize (*Zea mays* L.). Bot. Rev. 35 (2), 117–168. 10.1007/BF02858912

[B36] Chase S. S. (1952). Production of homozygous diploids of maize from monoploids ^1^ . Agron. J. 44 (5), 263–267. 10.2134/agronj1952.00021962004400050010x

[B37] Chen B. Y. Heneen W. K. (1989). Evidence for spontaneous diploid androgenesis in *Brassica napus* L. Sex. Plant Reprod. 2 (1), 15–17. 10.1007/BF00190114

[B38] Chen J.-M. Chuzhanova N. Stenson P. D. Férec C. Cooper D. N. (2005). Meta-Analysis of gross insertions causing human genetic disease: Novel mutational mechanisms and the role of replication slippage. Hum. Mutat. 25 (2), 207–221. 10.1002/humu.20133 15643617

[B39] Chen J. F. Cui L. Malik A. A. Mbira K. G. (2011). *In vitro* haploid and dihaploid production via unfertilized ovule culture, *Plant Cell, Tissue and Organ Culture* , 104, 311–319. 10.1007/s11240-010-9874-6

[B40] Chen Z. J. (2010). Molecular mechanisms of polyploidy and hybrid vigor. Trends Plant Sci. 15 (2), 57–71. 10.1016/j.tplants.2009.12.003 20080432 PMC2821985

[B41] Chester M. Gallagher J. P. Symonds V. V. da Silva A. V. C. Mavrodiev E. v. Leitch A. R. (2012). Extensive chromosomal variation in a recently formed natural allopolyploid species, *Tragopogon miscellus* (Asteraceae). Proc. Natl. Acad. Sci. U. S. A. 109 (4), 1176–1181. 10.1073/pnas.1112041109 22228301 PMC3268322

[B42] Chhuneja P. Kaur S. Goel R. K. Aghaee-Sarbarzeh M. Dhaliwal H. S. (2007). “Introgression of leaf rust and stripe rust resistance genes from Aegilops umbellulata to hexaploid wheat through induced homoeologous pairing,” in Wheat production in stressed environments. Editors Buck H. Nisi J. Salomón N. (Springer Netherlands), 83–90. 10.1007/1-4020-5497-1_10

[B43] Cho Y. B. Jones S. I. Vodkin L. O. (2019). Nonallelic homologous recombination events responsible for copy number variation within an RNA silencing locus. Plant direct 3 (8), e00162. 10.1002/pld3.162 31468028 PMC6710647

[B44] Choo T. M. Reinbergs E. Kasha K. J. (2011). Use of haploids in breeding barley. Plant Breed. Rev. 3, 219–252. 10.1002/9781118061008.ch5

[B45] Chung Y. S. Choi S. C. Jun T.-H. Kim C. (2017). Genotyping-by-sequencing: A promising tool for plant genetics research and breeding. Hortic. Environ. Biotechnol. 58 (5), 425–431. 10.1007/s13580-017-0297-8

[B46] Clapham D. (1973). Haploid *Hordeum* plants from anthers *in-vitro* . J. Plant Breed. 69 (2), 142–155. 10.1007/978-94-017-1856-1_5

[B47] Clausen R. E. Lammerts W. E. (1929). Interspecific hybridization in *nicotiana*. X. Haploid and diploid merogony. Am. Nat. 63 (686), 279–282. 10.1086/280261

[B48] Collins G. B. Grosser J. W. (1984). “Culture of embryos,” in Cell culture and somatic cell genetics of plants. Editor Vasil I. K. (Cambridge: Academic Press), 241–257.

[B49] Comai L. (2005). The advantages and disadvantages of being polyploid. Nat. Rev. Genet. 6 (11), 836–846. 10.1038/nrg1711 16304599

[B50] Constabel F. Cutler A. J. (1985). “Protoplast fusion,” in Plant protoplasts. Editors Fowke L. C. Constabel F., 53–65. 10.1201/9781351075770

[B51] Copenhaver G. Browne W. Preuss D. (1998). Assaying genome-wide recombination and centromere functions with Arabidopsis tetrads. Proc. Natl. Acad. Sci. U. S. A. 95 (1), 247–252. 10.1073/pnas.95.1.247 9419361 PMC18190

[B52] Datta S. K. Wenzel G. (1987). Isolated microspore derived plant formation via embryogenesis in *Triticum aestivum* L. Plant Sci. 48 (1), 49–54. 10.1016/0168-9452(87)90069-0

[B53] Defra (2021). The regulation of genetic technologies: A public consultation on the regulation of genetic technologies. Available at: www.gov.uk/government/publications 14 .

[B54] Dewey D. R. (1979). Some applications and misapplications of induced polyploidy to plant breeding. Basic Life Sci. 13, 445–470. 10.1007/978-1-4613-3069-1_23 550837

[B55] Dewitte A. Van K. Van J. (2012). “Use of 2n gametes in plant breeding,” in Plant breeding. Editor Abdurakhmonov I. Y. (Rijeka: IntechOpen, 58–89. 10.5772/29827

[B58] Dhooghe E. van Laere K. Eeckhaut T. Leus L. van Huylenbroeck J. (2011). Mitotic chromosome doubling of plant tissues *in vitro* . Plant Cell Tissue Organ Cult. 104 (3), 359–373. 10.1007/s11240-010-9786-5

[B59] Doll N. M. Gilles L. M. Gérentes M. F. Richard C. Just J. Fierlej Y. (2019). Single and multiple gene knockouts by CRISPR-Cas9 in maize. Plant Cell Rep. 38 (4), 487–501. 10.1007/s00299-019-02378-1 30684023

[B60] Dorsey E. (1936). Induced polyploidy in wheat and rye. J. Hered. 27 (4), 155–160. 10.1093/oxfordjournals.jhered.a104195

[B61] Drouaud J. Camilleri C. Bourguignon P.-Y. Canaguier A. Bérard A. Vezon D. (2006). Variation in crossing-over rates across chromosome 4 of *Arabidopsis thaliana* reveals the presence of meiotic recombination “hot spots”. Genome Res. 16 (1), 106–114. 10.1101/gr.4319006 16344568 PMC1356134

[B62] Dyer A. F. Jong K. Ratter J. A. (1970). Aneuploidy: A redefinition. Notes R. Botanic Gard. Edingurgh 30 (1), 177–182.

[B63] Edger P. P. Poorten T. J. VanBuren R. Hardigan M. A. Colle M. McKain M. R. (2019). Origin and evolution of the octoploid strawberry genome. Nat. Genet. 51 (3), 541–547. 10.1038/s41588-019-0356-4 30804557 PMC6882729

[B64] Ehrensberger R. (1948). Versuche zur Auslösung von Haploidie bei Blütenpflanzen. Biol. Zentralblatt 67, 537–546.

[B65] Entine J. Felipe M. S. S. Groenewald J.-H. Kershen D. L. Lema M. McHughen A. (2021). Regulatory approaches for genome edited agricultural plants in select countries and jurisdictions around the world. Transgenic Res. 30, 551–584. 10.1007/s11248-021-00257-8 33970411 PMC8316157

[B66] Fatokun C. A. (2000). “Breeding cowpea for resistance to insect pests: Attempted crosses between cowpea and vigna vexillata,” in Challenges and opportunities for enhancing sustainable cowpea production. Proceedings of the world cowpea conference III held at the international Institute of tropical Ag. Editors Fatokun C. . Tarawali S. A. Singh B. B. Kormawa P. M. Tamo M. (Ibadan, Nigeria: IITA), 52–61.

[B67] Fernandes J. B. Séguéla-Arnaud M. Larchevêque C. Lloyd A. H. Mercier R. (2018). Unleashing meiotic crossovers in hybrid plants. Proc. Natl. Acad. Sci. U. S. A. 115 (10), 2431–2436. 10.1073/pnas.1713078114 29183972 PMC5877974

[B68] Forster B. P. (2004). Mutation genetics of salt tolerance in barley: An assessment of Golden Promise and other semi-dwarf mutants. Euphytica 120, 317–328. 10.1023/A:1017592618298

[B69] Friebe B. Jiang J. Raupp W. J. McIntosh R. A. Gill B. S. (1996). Characterization of wheat-alien translocations conferring resistance to diseases and pests: Current status. Euphytica 91 (1), 59–87. 10.1007/BF00035277

[B70] Fuchs L. K. Jenkins G. Phillips D. W. (2018). Anthropogenic impacts on meiosis in plants. Front. Plant Sci. 9, 1429. 10.3389/fpls.2018.01429 30323826 PMC6172301

[B71] Funatsuki H. Suzuki M. Hirose A. Inaba H. Yamada T. Hajika M. (2014). Molecular basis of a shattering resistance boosting global dissemination of soybean. Proc. Natl. Acad. Sci. U. S. A. 111 (50), 17797–17802. 10.1073/pnas.1417282111 25468966 PMC4273335

[B72] Furman B. J. Qualset C. O. Skovmand B. Heaton J. H. Corke H. Wesenberg D. M. (1997). Characterization and analysis of north American triticale genetic resources. Crop Sci. 37 (6), 1951–1959. 10.2135/cropsci1997.0011183X003700060046x

[B73] Gao C. (2021). Genome engineering for crop improvement and future agriculture. Cell 184 (6), 1621–1635. 10.1016/j.cell.2021.01.005 33581057

[B74] Gao H. Mutti J. Young J. K. Yang M. Schroder M. Lenderts B. (2020). Complex trait loci in maize enabled by CRISPR-cas9 mediated gene insertion. Front. Plant Sci. 11, 535. 10.3389/fpls.2020.00535 32431725 PMC7214728

[B75] Gao L. Diarso M. Zhang A. Zhang H. Dong Y. Liu L. (2016). Heritable alteration of DNA methylation induced by whole-chromosome aneuploidy in wheat. New Phytol. 209 (1), 364–375. 10.1111/nph.13595 26295562

[B76] Gerassimova H. (1936). Experimentally produced haploid plant in *Crepis tectorum* . Biol. J. 5, 895–900.

[B77] Ghoshal B. Picard C. L. Vong B. Feng S. Jacobsen S. E. (2021). CRISPR-based targeting of DNA methylation in *Arabidopsis thaliana* by a bacterial CG-specific DNA methyltransferase. Proc. Natl. Acad. Sci. U. S. A. 118 (23), e2125016118. 10.1073/pnas.2125016118 34074795 PMC8201958

[B78] Gill B. S. Waines J. G. Sharma H. C. (1981). Endosperm abortion and the production of viable *Aegilops squarrosa* x *Triticum boeoticum* hybrids by embryo culture. Plant Sci. Lett. 23 (2), 181–187. 10.1016/0304-4211(81)90010-9

[B79] Giraut L. Falque M. Drouaud J. Pereira L. Martin O. C. Mézard C. (2011). Genome-wide crossover distribution in *Arabidopsis thaliana* meiosis reveals sex-specific patterns along chromosomes. PLoS Genet. 7 (11), e1002354. 10.1371/journal.pgen.1002354 22072983 PMC3207851

[B80] Glimelius K. Wallin A. Eriksson T. (1978). Concanavalin A improves the polyethylene glycol method for fusing plant protoplasts. Physiol. Plant. 44 (2), 92–96. 10.1111/j.1399-3054.1978.tb01620.x

[B81] Goering R. v. Pattee P. A. (1971). Mutants of *Staphylococcus aureus* with increased sensitivity to ultraviolet radiation. J. Bacteriol. 106 (1), 157–161. 10.1128/jb.106.1.157-161.1971 4251664 PMC248656

[B82] Goodsell S. F. (1961). Male sterility in corn by androgenesis ^1^ . Crop Sci. 1 (3), 227–228. 10.2135/cropsci1961.0011183x000100030022x

[B83] Green A. G. Dribnenki J. C. P. (1996). in Breeding and development of Linola™ (low linolenic flax)FAO-Proc.R.B.R. Group. 3rd intern ed. (Rome: FAO).

[B84] Griffiths A. J. Miller J. H. Suzuki D. T. Lewontin R. C. Gelbart W. M. (Editors) (2000). Induced mutations (New York: W. H. Freeman).

[B85] Grosser J. W. Gmitter F. G. (2011). Protoplast fusion for production of tetraploids and triploids: Applications for scion and rootstock breeding in citrus. Plant Cell Tissue Organ Cult. 104 (3), 343–357. 10.1007/s11240-010-9823-4

[B86] Guha S. Maheshwari S. C. (1966). Cell division and differentiation of embryos in the pollen grains of datura *in vitro* . Nature 212 (5057), 97–98. 10.1038/212097a0

[B87] Guha S. Maheshwari S. C. (1964). *In vitro* production of embryos from anthers of *datura* . Nature. 204 (4957), 497. 10.1038/204497a0

[B88] Hasan N. Choudhary S. Naaz N. Sharma N. Laskar R. A. (2021). Recent advancements in molecular marker-assisted selection and applications in plant breeding programmes. J. Genet. Eng. Biotechnol. 19 (1), 128. 10.1186/s43141-021-00231-1 34448979 PMC8397809

[B89] Haslam T. M. Yeung E. C. (2011). “Zygotic embryo culture: An overview,” in Plant embryo culture: Methods and protocols. Editors Thorpe T. A. Yeung E. C. (New York: Springer), 3–15. 10.1007/978-1-61737-988-8_1 21207257

[B90] Hegarty M. J. Barker G. L. Brennan A. C. Edwards K. J. Abbott R. J. Hiscock S. J. (2008). Changes to gene expression associated with hybrid speciation in plants: Further insights from transcriptomic studies in *Senecio* . Philos. Trans. R. Soc. Lond. B Biol. Sci. 363 (1506), 3055–3069. 10.1098/rstb.2008.0080 18579474 PMC2607317

[B91] Henry I. M. Dilkes B. P. Miller E. S. Burkart-Waco D. Comai L. (2010). Phenotypic consequences of aneuploidy in *Arabidopsis thaliana* . Genetics 186 (4), 1231–1245. 10.1534/genetics.110.121079 20876566 PMC2998307

[B92] Heslop-Harrison J. S. Schwarzacher T. (2007). Domestication, genomics and the future for banana. Ann. Bot. 100 (5), 1073–1084. 10.1093/aob/mcm191 17766312 PMC2759213

[B93] Hodgkinson A. Eyre-Walker A. (2011). Variation in the mutation rate across mammalian genomes. Nat. Rev. Genet. 12 (11), 756–766. 10.1038/nrg3098 21969038

[B94] Hoffmann G. R. Calciano M. A. Lawless B. M. Mahoney K. M. (2003). Frameshift mutations induced by three classes of acridines in the lacZ reversion assay in *Escherichia coli*: Potency of responses and relationship to slipped mispairing models. Environ. Mol. Mutagen. 42 (2), 111–121. 10.1002/em.10182 12929124

[B95] IAEA (2022). IAEA mutant database. Vienna: International Atomic Energy Agency. Available from: http://mvd.iaea.org/(accessed. April, 2022).

[B96] Jacobs M. (1969). Studies on the genetic activity of thymidine-base analogue in *Arabidopsis thaliana* . Mutat. Res. 7 (1), 51–62. 10.1016/0027-5107(69)90049-9 4240680

[B97] Jain S. M. (2001). Tissue culture-derived variation in crop improvement. Euphytica. 118 (2), 153–166. 10.1023/A:1004124519479

[B98] Jelesko J. G. Harper R. Furuya M. Gruissem W. (1999). Rare germinal unequal crossing-over leading to recombinant gene formation and gene duplication in *Arabidopsis thaliana* . Proc. Natl. Acad. Sci. U. S. A. 96 (18), 10302–10307. 10.1073/pnas.96.18.10302 10468603 PMC17883

[B99] Jo K.-R. Kim C.-J. Kim S.-J. Kim T.-Y. Bergervoet M. Jongsma M. A. (2014). Development of late blight resistant potatoes by cisgene stacking. BMC Biotechnol. 14 (1), 50. 10.1186/1472-6750-14-50 24885731 PMC4075930

[B100] Jones H. D. (2015a). Challenging regulations: Managing risks in crop biotechnology. Food Energy secur. 4 (2), 87–91. 10.1002/fes3.60 27867501 PMC5111418

[B101] Jones H. D. (2015b). Regulatory uncertainty over genome editing. Nat. Plants 1, 14011. 10.1038/nplants.2014.11 27246057

[B102] Joyce S. M. Cassells A. C. Mohan Jain S. (2003). Stress and aberrant phenotypes *in vitro* culture. Plant Cell Tissue Organ Cult. 74 (2), 103–121. 10.1023/A:1023911927116

[B103] Jung W. J. Seo Y. W. (2014). Employment of wheat-rye translocation in wheat improvement and broadening its genetic basis. J. Crop Sci. Biotechnol. 17 (4), 305–313. 10.1007/s12892-014-0086-1

[B104] Kameya T. (1975). Induction of hybrids through somatic cell fusion with dextran sulfate and gelatin. Jpn. J. Genet. 50 (3), 235–246. 10.1266/jjg.50.235

[B105] Kao K. N. Michayluk M. R. (1974). A method for high-frequency intergeneric fusion of plant protoplasts. Planta 115 (4), 355–367. 10.1007/BF00388618 24458930

[B106] Kao K. N. Saleem M. Abrams S. Pedras M. Horn D. Mallard C. (1991). Culture conditions for induction of green plants from barley microspores by anther culture methods. Plant Cell Rep. 9 (11), 595–601. 10.1007/BF00231796 24213657

[B107] Kasha K. J. Kao K. N. (1970). High frequency haploid production in barley (*Hordeum vulgare* L.). Nature 225 (5235), 874–876. 10.1038/225874a0 16056782

[B108] Kasha K. J. Maluszynski M. (2003). “Production of doubled haploids in crop plants. An introduction,” in Doubled haploid production in crop plants (Springer Netherlands), 1–4. 10.1007/978-94-017-1293-4_1

[B109] Katepa-Mupondwa F. M. Christie B. R. Michaels T. E. (2002). An improved breeding strategy for autotetraploid alfalfa (*Medicago sativa* L.). Euphytica 123 (1), 139–146. 10.1023/A:1014488307000

[B110] Keeney S. Giroux C. N. Kleckner N. (1997). Meiosis-specific DNA double-strand breaks are catalyzed by Spo11, a member of a widely conserved protein family. Cell 88 (3), 375–384. 10.1016/S0092-8674(00)81876-0 9039264

[B111] Kehr A. E. (1951). Monoploidy in nicotiana. J. Hered. 42 (2), 107–112. 10.1093/oxfordjournals.jhered.a106160 14832463

[B112] Kermicle J. (1974). Haploids in higher plants: Advances and potential. Guelph, Canada: University of Guelph. 10.1007/978-1-4612-2694-9_58 Origin of androgenetic haploids and diploids induced by the indeterminate gametophyte (ig) mutation in maize

[B113] Kharkwal M. C. Shu Q. Y. (2009). “The role of induced mutations in world food security,” in Induced plant mutations in the genomics era proceedings of an international joint FAO/IAEA symposium.

[B114] Khush G. S. (1973). Cytogenetics of aneuploids. New York, N.Y: Acad.Press. 10.1016/b978-0-12-406250-4.x5001-6

[B115] Kim Y. S. Schumaker K. S. Zhu J. K. (2006). EMS mutagenesis of Arabidopsis. Methods Mol. Biol. 323, 101–103. 10.1385/1-59745-003-0:101 16739570

[B116] Kimber G. (1977). “The use of aneuploids in studies of genetics, breeding, and evolution in wheat,” in Genetic diversity in plants. Editors Muhammed A. Aksel R. von Borstel R. C. (Boston, MA: Springer US), 103–116. 10.1007/978-1-4684-2886-5_11 1073205

[B117] Kostoff D. (1929). An androgenic *nicotiana* haploid. Z. Zellforsch. 9 (4), 640–642. 10.1007/BF02450775

[B118] Kovalchuk I. Kovalchuk O. Hohn B. (2000). Genome-wide variation of the somatic mutation frequency in transgenic plants. EMBO J. 19 (17), 4431–4438. 10.1093/emboj/19.17.4431 10970837 PMC302052

[B119] Łapiński B. (2002). “Application of tetraploid triticale in improvement of related crop species,” in Proceedings of the 5th International Triticale Symposium, Radzików, Poland, 30 June - 5 July, 71–77. Volume I: oral presentations.

[B120] Laurie D. A. Bennett M. D. (1988). The production of haploid wheat plants from wheat x maize crosses. Theor. Appl. Genet. 76 (3), 393–397. 10.1007/BF00265339 24232203

[B121] Lawrence E. J. Griffin C. H. Henderson I. R. (2017). Modification of meiotic recombination by natural variation in plants. J. Exp. Bot. 68 (20), 5471–5483. 10.1093/jxb/erx306 28992351

[B122] Lentini Z. Restrepo G. Buitrago M. E. Tabares E. (2020). Protocol for rescuing young cassava embryos. Front. Plant Sci. 11, 522. 10.3389/fpls.2020.00522 32457774 PMC7227409

[B123] Lercher M. J. Hurst L. D. (2002). Human SNP variability and mutation rate are higher in regions of high recombination. Trends Genet. 18 (7), 337–340. 10.1016/S0168-9525(02)02669-0 12127766

[B124] Levin D. (2002). The role of chromosomal change in plant evolution. Oxf. Ser. Ecol. Abd Evol. 1 (7), 241. 10.1600/036364404774195656

[B125] Li J. Meng X. Zong Y. Chen K. Zhang H. Liu J. (2016). Gene replacements and insertions in rice by intron targeting using CRISPR-Cas9. Nat. Plants 2, 16139. 10.1038/nplants.2016.139 27618611

[B126] Li J. Sun Y. Du J. Zhao Y. Xia L. (2017). Generation of targeted point mutations in rice by a modified CRISPR/Cas9 system. Mol. Plant 10 (3), 526–529. 10.1016/j.molp.2016.12.001 27940306

[B127] Li S. Lin D. Zhang Y. Deng M. Chen Y. Lv B. (2022). Genome-edited powdery mildew resistance in wheat without growth penalties. Nature 602 (7897), 455–460. 10.1038/s41586-022-04395-9 35140403

[B128] Li S. Zheng Y-C. Shu Q-Y. Cui H-R. Fu H-W. Huang J-Z. (2016). Frequency and type of inheritable mutations induced by γ rays in rice as revealed by whole genome sequencing. J. Zhejiang Univ. Sci. B 17 (12), 905–915. 10.1631/jzus.B1600125 27921396 PMC5172596

[B129] Liu J. Xu X. Deng X. (2005). Intergeneric somatic hybridization and its application to crop genetic improvement. Plant Cell Tissue Organ Cult. 82 (1), 19–44. 10.1007/s11240-004-6015-0

[B130] Lundin C. North M. Erixon K. Walters K. Jenssen D. Goldman A. S. H. (2012). Methyl methanesulfonate (MMS) produces heat-labile DNA damage but no detectable *in vivo* DNA double-strand breaks. Nucleic Acids Res. 40 (12), 5794. 10.1093/nar/gks589 PMC1174933 16009812

[B131] Magni G. E. von Borstel R. C. (1962). Different rates of spontaneous mutation during mitosis and meiosis in yeast. Genetics 47 (8), 1097–1108. 10.1093/genetics/47.8.1097 17248123 PMC1210391

[B132] Mallapaty S. (2019). Australian gene-editing rules adopt ‘middle ground’. Nature. 10.1038/d41586-019-01282-8 32317780

[B133] Mallikarjuna N. Jadhav D. Clarke H. Coyne C. Muehlbauer F. (2005). Induction of androgenesis as a consequence of wide crossing in chickpea. Sat. EJournal 1 (1), 1–3.

[B134] Martínez-Fortún J. Phillips D. W. Jones H. D. (2017). Potential impact of genome editing in world agriculture. Emerg. Top. Life Sci. 1 (2), 117–133. 10.1042/ETLS20170010 33525764

[B135] Masterson J. (1994). Stomatal size in fossil plants: Evidence for polyploidy in majority of angiosperms. Science 264, 421–424. 10.1126/science.264.5157.421 17836906

[B136] Mazia D. (1987). The chromosome cycle and the centrosome cycle in the mitotic cycle. Int. Rev. Cytol. 100 (C), 49–92. 10.1016/S0074-7696(08)61698-8 3549609

[B137] Mcgoverin C. M. Snyders F. Muller N. Botes W. Fox G. Manley M. (2011). A review of triticale uses and the effect of growth environment on grain quality. J. Sci. Food Agric. 91 (7), 1155–1165. 10.1002/jsfa.4338 21433010

[B138] McNaughton I. H. (1973). Synthesis and sterility of *raphanobrassica* . Euphytica 22 (1), 70–88. 10.1007/BF00021558

[B139] Melchers G. Labib G. (1974). Somatic hybridisation of plants by fusion of protoplasts. Molec. Gen. Genet. 135 (4), 277–294. 10.1007/BF00271144

[B140] Mendoza H. A. Haynes F. L. (1974). Genetic basis of heterosis for yield in the autotetraploid potato. Theor. Appl. Genet. 45 (1), 21–25. 10.1007/BF00281169 24419217

[B141] Menz J. Modrzejewski D. Hartung F. Wilhelm R. Sprink T. (2020). Genome edited crops touch the market: A view on the global development and regulatory environment. Front. Plant Sci. 11 (1525), 586027. 10.3389/fpls.2020.586027 33163013 PMC7581933

[B142] Mercé C. Bayer P. E. Tay Fernandez C. Batley J. Edwards D. (2020). Induced methylation in plants as a crop improvement tool: Progress and perspectives. Agronomy 10 (10), 1484. 10.3390/AGRONOMY10101484

[B143] Merker A. (1975). Chromosome composition of hexaploid triticale. Hereditas 80 (1), 41–52. 10.1111/j.1601-5223.1975.tb01498.x

[B144] Mertz T. M. Harcy V. Roberts S. A. (2017). Risks at the DNA replication fork: Effects upon carcinogenesis and tumor heterogeneity. Genes 8 (1), 46. 10.3390/genes8010046 PMC5295039 28117753

[B145] Michel W. (1938). Über die experimentelle Fusion pflanzlicher Protoplasten. Protoplasma 30, 471. 10.1007/bf01613814

[B146] Miller T. E. Reader S. M. Ainsworth C. C. Summers R. W. C. N.-P. (1987). “The introduction of a major gene for resistance to powdery midew of wheat, erysiphe graminis F. Sp. Tritici, from Aegilops speltoides into wheat, Triticum aestivum,” in Proceedings of the EUCARPIA conference cereal breeding related to integrated cereal production. Editors Jorna M. L. Shootmaker L. A. J. (Wageningen), 179–183.

[B147] Minoia S. Petrozza A. D’Onofrio O. Piron F. Mosca G. Sozio G. (2010). A new mutant genetic resource for tomato crop improvement by TILLING technology. BMC Res. Notes 3, 69. 10.1186/1756-0500-3-69 20222995 PMC2845601

[B148] Molla K. A. Sretenovic S. Bansal K. C. Qi Y. (2021). Precise plant genome editing using base editors and prime editors. Nat. Plants 7 (9), 1166–1187. 10.1038/s41477-021-00991-1 34518669

[B149] Moody M. E. Mueller L. D. Soltis D. E. (1993). Genetic variation and random drift in autotetraploid populations. Genetics 134 (2), 649–657. 10.1093/genetics/134.2.649 8325493 PMC1205504

[B150] Novak F. J. Brunner H. (1992). Plant breeding: Induced mutation technology for crop improvement. IAEA Bull. 24 (5), 25–33.

[B151] Nagata T. (1978). A novel cell-fusion method of protoplasts by polyvinyl alcohol. Naturwissenschaften 65, 263–264. 10.1007/BF00368577

[B152] Nagata T. Eibl H. Melchers G. (1979). Fusion of plant protoplasts induced by a positively charged synthetic phospholipid. Zeitschrift Fur Naturforschung - Sect. C J. Biosci. 34 (5–6), 460–462. 10.1515/znc-1979-5-624

[B153] Najera V. A. Twyman R. M. Christou P. Zhu C. (2019). Applications of multiplex genome editing in higher plants. Curr. Opin. Biotechnol. 59, 93–102. 10.1016/j.copbio.2019.02.015 30978482

[B154] Oettler G. (2005). The fortune of a botanical curiosity - triticale: Past, present and future. J. Agric. Sci. 143 (5), 329–346. 10.1017/S0021859605005290

[B155] Oettler G. Wehmann F. Utz H. F. (1991). Influence of wheat and rye parents on agronomic characters in primary hexaploid and octoploid triticale. Theor. Appl. Genet. 81 (3), 401–405. 10.1007/BF00228683 24221272

[B156] Oladosu Y. Rafii M. Y. Abdullah N. Hussin G. Ramli A. Rahim H. A. (2016). Principle and application of plant mutagenesis in crop improvement: A review. Biotechnol. Biotechnol. Equip. 30 (1), 1–16. 10.1080/13102818.2015.1087333

[B157] Olsen R. T. Ranney T. G. Viloria Z. (2006). Reproductive behavior of induced allotetraploid x Chitalpa and *in vitro* embryo culture of polyploid progeny. J. Am. Soc. Hortic. Sci. 131 (6), 716–724. 10.21273/jashs.131.6.716

[B158] Osborn T. C. Pires J. C. Birchler J. A. Auger D. L. Chen Z. J. Lee H.-S. (2003). Understanding mechanisms of novel gene expression in polyploids. Trends Genet. 19 (3), 141–147. 10.1016/s0168-9525(03)00015-5 12615008

[B159] Ossowski S. Schneeberger K. Lucas-Lledó J. I. Warthmann N. Clark R. M. Shaw R. G. (2010). The rate and molecular spectrum of spontaneous mutations in *Arabidopsis thaliana* . Science 327 (5961), 92–94. 10.1126/science.1180677 20044577 PMC3878865

[B160] Ouyang T.-W. Hu H. Chuang C.-C. Tseng C.-C. (1973). Induction of pollen plants from anthers of *Triticum aestivum* L. Cultured *in vitro* . Sci. Sin. 16 (1), 79–90. 10.1360/ya1973-16-1-79

[B161] Ovcharenko O. Momot V. Cherep N. Sheludko Y. Komarnitsky I. Rudas V. (2011). Transfer of transformed *Lesquerella fendleri* (Gray) Wats. chloroplasts into *Orychophragmus violaceus* (L.) O.E. Schulz by protoplast fusion. Plant Cell Tissue Organ Cult. 105 (1), 21–27. 10.1007/s11240-010-9833-2

[B162] Pelletier G. Primard C. Vedel F. Chetrit P. Remy R. Rousselle P. (1983). Intergeneric cytoplasmic hybridization in *Cruciferae* by protoplast fusion. Mol. Gen. Genet. 191 (2), 244–250. 10.1007/BF00334821

[B163] Peloquin S. J. Boiteux L. S. Carputo D. (1999). Meiotic mutants in potato: Valuable variants. Genetics 153 (4), 1493–1499. 10.1093/genetics/153.4.1493 10581260 PMC1460881

[B164] Perry J. Ashworth A. (1999). Evolutionary rate of a gene affected by chromosomal position. Curr. Biol. 9 (17), 987–989. 10.1016/S0960-9822(99)80430-8 10508587

[B165] Pichot C. el Maâtaoui M. Raddi S. Raddi P. (2001). Surrogate mother for endangered Cupressus. Nature 412 (6842), 39. 10.1038/35083687 11452293

[B166] Pichot C. El Maâtaoui M. (2000). Unreduced diploid nuclei in *Cupressus dupreziana* A. Camus pollen. Theor. Appl. Genet. 101 (4), 574–579. 10.1007/s001220051518

[B168] Pichot C. Liens B. Rivera Nava J. L. Bachelier J. B. El Maâtaoui M. (2008). Cypress surrogate mother produces haploid progeny from alien pollen. Genetics 178 (1), 379–383. 10.1534/genetics.107.080572 18202380 PMC2206086

[B169] Pigneur L. M. Hedtke S. M. Etoundi E. van Doninck K. (2012). Androgenesis: A review through the study of the selfish shellfish *corbicula* spp. Heredity 108 (6), 581–591. 10.1038/hdy.2012.3 22473310 PMC3356815

[B170] Pitsikas P. Patapas J. M. Cupples C. G. (2004). Mechanism of 2-aminopurine-stimulated mutagenesis in *Escherichia coli* . Mutat. Res. 550 (1–2), 25–32. 10.1016/j.mrfmmm.2004.01.008 15135638

[B171] Planchais S. Glab N. Inzé D. Bergounioux C. (2000). Chemical inhibitors: A tool for plant cell cycle studies. FEBS Lett. 476 (1–2), 78–83. 10.1016/S0014-5793(00)01675-6 10878255

[B172] Podevin N. Davies H. V. Hartung F. Nogué F. Casacuberta J. M. (2013). Site-directed nucleases: A paradigm shift in predictable, knowledge-based plant breeding. Trends Biotechnol. 31 (6), 375–383. 10.1016/j.tibtech.2013.03.004 23601269

[B173] Power J. B. Cummins S. E. Cocking E. C. (1970). Fusion of isolated plant protoplasts. Nature 225 (5237), 1016–1018. 10.1038/2251016a0 16056900

[B174] Pratto F. Brick K. Khil P. Smagulova F. Petukhova G. v. Camerini-Otero D. (2014). DNA recombination. Recombination initiation maps of individual human genomes, Science 346 (6211), 1256442. 10.1126/science.1256442 25395542 PMC5588152

[B175] Prieto P. (2020). Chromosome manipulation for plant breeding purposes. Agronomy 10 (11), 1695. 10.3390/agronomy10111695

[B176] Quesneville H. (2020). Twenty years of transposable element analysis in the *Arabidopsis thaliana* genome. Mob. DNA 11 (1), 28. 10.1186/s13100-020-00223-x 32742313 PMC7385966

[B177] Reed S. M. (2005). “Embryo rescue,” in Plant development and biotechnology. Editors Robert N. Trigiano R. N. Gray D. J. (Boca Raton: CRC Press), 235–240.

[B178] Rabinovich S. v. (1998). Importance of wheat-rye translocations for breeding modern cultivars of *Triticum aestivum* L. Euphytica 100 (1–3), 323–340. 10.1023/a:1018361819215

[B179] Rajaram S. Mann C. H. E. Qrtiz-Ferrara G. Mujeeb-Kazi A. (1983). “Adaptation, stability and high yield potential of certain 1B/1R CIMMYT wheats,” in Proc. 6th Int. Wheat. Genet. Symp., 613–621.

[B180] Ram M. (2014). “Polyploidy and distant hybridization in plant breeding,” in Plant breeding methods. Editor Ram M. (New Delhi: PHI Learning Pvt Ltd), 423–445.

[B181] Ramanna M. S. Jacobsen E. (2003). Relevance of sexual polyploidization for crop improvement - a review. Euphytica 133 (1), 3–8. 10.1023/A:1025600824483

[B182] Rambaud C. Dubois J. Vasseur J. (1992). The induction of tetraploidy in chicory (Cichorium intybus L. var. Magdebourg) by protoplast fusion. Euphytica 62 (1), 63–67. 10.1007/BF00036089

[B183] Ramming D. W. (2019). The use of embryo culture in fruit breeding. HortScience 25 (4), 393–398. 10.21273/hortsci.25.4.393

[B184] Ramsey J. Schemske D. W. (1998). Pathways, mechanisms, and rates of polyploid formation in flowering plants. Annu. Rev. Ecol. Syst. 29, 467–501. 10.1146/annurev.ecolsys.29.1.467

[B185] Randolph L. F. (1932). Some effects of high temperature on polyploidy and other variations in maize. Proc. Natl. Acad. Sci. U. S. A. 18 (3), 222–229. 10.1073/pnas.18.3.222 16587665 PMC1076195

[B186] Randolph L. F. (1942). The influence of heterozygosis on fertility and vigor in autotetraploid maize. Genetics 27, 163.

[B187] Rattray A. Santoyo G. Shafer B. Strathern J. N. (2015). Elevated mutation rate during meiosis in *Saccharomyces cerevisiae* . PLoS Genet. 11 (1), e1004910. 10.1371/journal.pgen.1004910 25569256 PMC4287439

[B188] Ravi M. Chan S. W. L. (2010). Haploid plants produced by centromere-mediated genome elimination. Nature 464 (7288), 615–618. 10.1038/nature08842 20336146

[B190] Renny-Byfield S. Wendel J. F. (2014). Doubling down on genomes: Polyploidy and crop plants. Am. J. Bot. 101 (10), 1711–1725. 10.3732/ajb.1400119 25090999

[B191] Rice genomes project (2014). The 3,000 rice genomes project. GigaScience 3 (7). 10.1186/2047-217X-3-7 PMC4035669 24872877

[B192] Rieger R. Michaelis A. Green M. (2012). Glossary of genetics and cytogenetics: Classical and molecular. Springer Berlin Heidelberg.

[B193] Roca Paixão J. F. Gillet F.-X. Ribeiro T. P. Bournaud C. Lourenço-Tessutti I. T. Noriega D. D. (2019). Improved drought stress tolerance in Arabidopsis by CRISPR/dCas9 fusion with a Histone AcetylTransferase. Sci. Rep. 9 (1), 8080. 10.1038/s41598-019-44571-y 31147630 PMC6542788

[B194] Ross-Ibarra J. Morrell P. L. Gaut B. S. (2007). Plant domestication, a unique opportunity to identify the genetic basis of adaptation. Proc. Natl. Acad. Sci. U. S. A. 104 (1), 8641–8648. 10.1073/pnas.0700643104 17494757 PMC1876441

[B195] Rosyara U. Kishii M. Payne T. Sansaloni C. P. Singh R. P. Braun H.-J. (2019). Genetic contribution of synthetic hexaploid wheat to CIMMYT’s spring bread wheat breeding germplasm. Sci. Rep. 9 (1), 12355. 10.1038/s41598-019-47936-5 31451719 PMC6710277

[B196] Roychowdhury R. Tah J. (2013). “Mutagenesis-a potential approach for crop improvement,” in Crop improvement: New approaches and modern techniques (Springer US), 149–187.

[B197] Salomé P. A. Bomblies K. Fitz J. Laitinen R. A. E. Warthmann N. Yant L. (2012). The recombination landscape in *Arabidopsis thaliana* F2 populations. Heredity 108 (4), 447–455. 10.1038/hdy.2011.95 22072068 PMC3313057

[B198] Sattler M. C. Carvalho C. R. Clarindo W. R. (2016). The polyploidy and its key role in plant breeding. Planta 243 (2), 281–296. 10.1007/s00425-015-2450-x 26715561

[B199] Schieder O. (1982). “Somatic hybridization: A new method for plant improvement,” in Plant improvement and somatic cell genetics. Editors Vasil I. K. Scowcroft W. R. Frey K. J. (Academic Press), 239–253.

[B200] Schifino Wittmann M. T. Dall’ Agnol M. (2003). Induçao de poliploidia No melhoramiento de plantas. Pesqui. Agropecuária Gaúcha 9 (1–2), 155–164. 10.29372/rab201917

[B201] Schmid M. W. Heichinger C. Coman Schmid D. Guthörl D. Gagliardini V. Bruggmann R. (2018). Contribution of epigenetic variation to adaptation in Arabidopsis. Nat. Commun. 9 (1), 4446. 10.1038/s41467-018-06932-5 30361538 PMC6202389

[B202] Schmidt C. Schindele P. Puchta H. (2020). From gene editing to genome engineering: Restructuring plant chromosomes via CRISPR/cas. aBIOTECH 1 (1), 21–31. 10.1007/s42994-019-00002-0 PMC9584095 36305002

[B203] Schmidt S. M. Belisle M. Frommer W. B. (2020). From gene editing to genome engineering: Restructuring plant chromosomes via CRISPR/cas. EMBO Rep. 21 (6), e50680. 10.15252/embr.202050680 32431018 PMC7271327

[B204] Schnable P. S. Ware D. Fulton R. S. Stein J. C. Wei F. Pasternak S. (2009). The B73 maize genome: Complexity, diversity, and dynamics. Science 326 (5956), 1112–1115. 10.1126/science.1178534 19965430

[B205] Schwander T. Oldroyd B. P. (2016). Androgenesis: Where males hijack eggs to clone themselves. Philos. Trans. R. Soc. Lond. B Biol. Sci. 371 (1706), 20150534. 10.1098/rstb.2015.0534 27619698 PMC5031619

[B206] Sears E. R. Gustafson J. P. (1993). Use of radiation to transfer alien chromosome segments to wheat. Crop Sci. 33 (5), 897–901. 10.2135/cropsci1993.0011183X003300050004x

[B207] Sears E. R. (1954). The aneuploids of common wheat. Missouri: College of Agriculture, Agricultural Experiment Station. University of Missouri.

[B208] Sears E. R. (1956). “The transfer of leaf-rust resistance from *Aegilops umbellulata* to wheat,” in Genetics in plant breeding (New York: Brook-haven Symposia in Biology), 1–22.

[B209] Seguí-Simarro J. M. (2010). Androgenesis revisited. Bot. Rev. 76 (3), 377–404. 10.1007/s12229-010-9056-6

[B210] Seguí-Simarro J. M. Corral-Martínez P. Parra-Vega V. González-García B. (2011). Androgenesis in recalcitrant solanaceous crops. Plant Cell Rep. 30 (5), 765–778. 10.1007/s00299-010-0984-8 21191595

[B211] Sencia M. Takeda J. Abe S. Nakamura T. (1979). Induction of cell fusion of plant protoplasts by electrical stimulation. Plant Cell Physiol. 20 (7), 1441–1443. 10.1093/oxfordjournals.pcp.a075944

[B212] Séré D. Martin A. (2020). Epigenetic regulation: Another layer in plant nutrition. Plant Signal. Behav. 15 (1), 1686236. 10.1080/15592324.2019.1686236 31674259 PMC7012064

[B213] Sharma C. B. S. R. (1990). Chemically induced aneuploidy in higher plants. Mutagenesis 5 (2), 105–125. 10.1093/mutage/5.2.105 2188063

[B215] Sharma D. R. Kaur R. Kumar K. (1996). Embryo rescue in plants - a review. Euphytica 89 (3), 325–337. 10.1007/BF00022289

[B216] Sheltzer J. M. Amon A. (2011). The aneuploidy paradox: Costs and benefits of an incorrect karyotype. Trends Genet. 27 (11), 446–453. 10.1016/j.tig.2011.07.003 21872963 PMC3197822

[B217] Silverman J. A. Oliver N. Andrew T. Tongchuan L. I. (2001). Resistance studies with daptomycin. Antimicrob. Agents Chemother. 45 (6), 1799–1802. 10.1128/AAC.45.6.1799-1802.2001 11353628 PMC90548

[B218] Soltis P. S. Soltis D. E. (2000). The role of genetic and genomic attributes in the success of polyploids. Proc. Natl. Acad. Sci. U. S. A. 97 (13), 7051–7057. 10.1073/pnas.97.13.7051 10860970 PMC34383

[B219] Stadler L. J. (1928a). Genetic effects of X-rays in maize. Proc. Natl. Acad. Sci. U. S. A. 14 (1), 69–75. 10.1073/pnas.14.1.69 16587308 PMC1085350

[B220] Stadler L. J. (1928b). Mutations in barley induced by X-rays and radium. Science 68 (1756), 186–187. 10.1126/science.68.1756.186 17774921

[B221] Stadler L. J. (1930). Some genetic effects of X-rays. J. Hered. 21 (1), 3–20. 10.1093/oxfordjournals.jhered.a103249

[B222] Stebbins G. L. (1971). Adaptive radiation of reproductive characteristics in angiosperms, II: Seeds and seedlings. Annu. Rev. Ecol. Syst. 2 (1), 237–260. 10.1146/annurev.es.02.110171.001321

[B223] Täckholm G. (1922). Zytologische Studien über die Gattung Rosa. Acta horti Bergiani 7, 97–381. 10.5962/bhl.title.15507

[B224] Torres E. M. Williams B. R. Amon A. (2008). Aneuploidy: Cells losing their balance. Genetics 179 (2), 737–746. 10.1534/genetics.108.090878 18558649 PMC2429870

[B225] Tsuda M. Watanabe K. N. Ohsawa R. (2019). Regulatory status of genome-edited organisms under the Japanese Cartagena Act. Front. Bioeng. Biotechnol. 7, 387. 10.3389/fbioe.2019.00387 31867318 PMC6908812

[B226] Valleau W. D. (1949). The genetics of mosaic resistance in *Nicotiana glutinosa* . J. Agric. Res. 78 (3–4), 77–79. https://pubmed.ncbi.nlm.nih.gov/18113660/. 18113660

[B227] Varotto S. Tani E. Abraham E. Krugman T. Kapazoglou A. Melzer R. (2020). Epigenetics: Possible applications in climate-smart crop breeding. J. Exp. Bot. 71 (17), 5223–5236. 10.1093/jxb/eraa188 32279074 PMC7475248

[B228] Veitia R. A. Bottani S. Birchler J. A. (2008). Cellular reactions to gene dosage imbalance: Genomic, transcriptomic and proteomic effects. Trends Genet. 24 (8), 390–397. 10.1016/j.tig.2008.05.005 18585818

[B229] Waldman A. S. (2008). Ensuring the fidelity of recombination in mammalian chromosomes. BioEssays 30 (11–12), 1163–1171. 10.1002/bies.20845 18937366

[B230] Wallin A. Glimelius K. Eriksson T. (1974). The induction of aggregation and fusion of *Dancus carota* protoplasts by polyethylene glycol. Z. für Pflanzenphysiol. 74, 64–80. 10.1016/S0044-328X(74)80205-9

[B231] Waltz E. (2022). GABA-enriched tomato is first CRISPR-edited food to enter market. Nat. Biotechnol. 40, 9–11. 10.1038/d41587-021-00026-2 34907351

[B232] Wang G. X. Tang Y. Yan H. Sheng X. G. Hao W. W. Zhang L. (2011). Production and characterization of interspecific somatic hybrids between *Brassica oleracea* var. botrytis and *B. nigra* and their progenies for the selection of advanced pre-breeding materials. Plant Cell Rep. 30 (10), 1811–1821. 10.1007/s00299-011-1088-9 21603996

[B233] Wang J. Jiang J. Wang Y. (2013). Protoplast fusion for crop improvement and breeding in China. Plant Cell Tissue Organ Cult. 112 (2), 131–142. 10.1007/s11240-012-0221-y

[B234] Wang M. van Bergen S. van Duijn B. (2000). Insights into a key developmental switch and its importance for efficient plant breeding. Plant Physiol. 124 (2), 523–530. 10.1104/pp.124.2.523 11027703 PMC1539284

[B235] Wang N. Yuan Y. Wang H. Yu D. Liu Y. Zhang A. (2020). Applications of genotyping-by-sequencing (GBS) in maize genetics and breeding. Sci. Rep. 10 (1), 16308. 10.1038/s41598-020-73321-8 33004874 PMC7530987

[B236] Weyen J. (2008). “Barley and wheat doubled haploids in breeding,” in Advances in haploid production in higher plants, 179–187. 10.1007/978-1-4020-8854-4_15

[B237] Wijnker E. James G. V. Ding J. Becker F. Klasen J. R. Rawat V. (2013). The genomic landscape of meiotic crossovers and gene conversions in *Arabidopsis thaliana* . eLife 2, e01426. 10.7554/eLife.01426 24347547 PMC3865688

[B238] Withers L. A. Cocking E. C. (1972). Fine-structural studies on spontaneous and induced fusion of higher plant protoplasts. J. Cell Sci. 11, 59–75. 10.1242/jcs.11.1.59 4341992

[B239] Wu Y. Sun Y. Sun S. Li G. Wang J. Wang B. (2018). Aneuploidization under segmental allotetraploidy in rice and its phenotypic manifestation. Theor. Appl. Genet. 131 (6), 1273–1285. 10.1007/s00122-018-3077-7 29478186 PMC5945760

[B240] Wyatt M. D. Pittman D. L. (2006). Methylating agents and DNA repair responses: Methylated bases and sources of strand breaks. Chem. Res. Toxicol. 19 (12), 1580–1594. 10.1021/tx060164e 17173371 PMC2542901

[B241] Xia G. (2009). Progress of chromosome engineering mediated by asymmetric somatic hybridization. J. Genet. Genomics 36 (9), 547–556. 10.1016/S1673-8527(08)60146-0 19782956

[B242] Xiong Z. Gaeta R. T. Pires J. C. (2011). Homoeologous shuffling and chromosome compensation maintain genome balance in resynthesized allopolyploid *Brassica napus* . Proc. Natl. Acad. Sci. U. S. A. 108 (19), 7908–7913. 10.1073/pnas.1014138108 21512129 PMC3093481

[B243] Yan H. Lu L. Alzate A. Ceballos H. Hershey C. Chen S. (2015). Fruit, seed and embryo development of different cassava (*Manihot esculenta* Crantz) genotypes and embryo rescue. Afr. J. Biotechnol. 13 (14), 1524–1528. 10.5897/ajb2013.13180

[B244] Yang N. Xu X. W. Wang R. R. Peng W. L. Cai L. Song J. M. (2017). Contributions of *Zea mays* subspecies *mexicana* haplotypes to modern maize. Nat. Commun. 8 (1), 1874. 10.1038/s41467-017-02063-5 29187731 PMC5707364

[B245] Yang S. Wang L. Huang J. Zhang X. Yuan Y. Chen J.-Q. (2015). Parent–progeny sequencing indicates higher mutation rates in heterozygotes. Nature 523 (7561), 463–467. 10.1038/nature14649 26176923

[B246] Zeller F. J. (1973). “1B/1R wheat-rye chromosome substitutions and translocations,” in Proceedings 4th international wheat genetics symposium. Editors Sears E. R. Sears L. M. S. (Missouri: Columbia: University of Missouri).

[B247] Zeller F. J. Hsam S. L. K. (1983). “Broadening the genetic variability of cultivated wheat by utilizing rye chromatin,” in Proceedings 6th international wheat genetics symposium. Editor Sakamoto S. (Kyoto, Japan: Kyoto: Kyoto University).

[B248] Zhang H. Bian Y. Gou X. Zhu B. Xu C. Qi B. (2013). Persistent whole-chromosome aneuploidy is generally associated with nascent allohexaploid wheat. Proc. Natl. Acad. Sci. U. S. A. 110 (9), 3447–3452. 10.1073/pnas.1300153110 23401544 PMC3587266

[B249] Zhang J. Stewart J. M. Turley R. B. (1999). “Analysis of semigamy expression in cotton (Gossypium barbadense),”. Editors Richter C. Dugger P. (Orlando: Beltwide Cotton Conferences), 446–447.Proceedings of the 1999 Beltwide Cotton Conference

[B250] Zhang Y. Massel K. Godwin I. D. Gao C. (2018). Applications and potential of genome editing in crop improvement. Genome Biol. 19 (1), 210. 10.1186/s13059-018-1586-y 30501614 PMC6267055

[B251] Zimmermann U. Scheurich P. (1981). High frequency fusion of plant protoplasts by electric fields. Planta 151, 26–32. 10.1007/BF00384233 24301666

